# Targeting the EGFR and Spindle Assembly Checkpoint Pathways in Oral Cancer: A Plausible Alliance to Enhance Cell Death

**DOI:** 10.3390/cancers16223732

**Published:** 2024-11-05

**Authors:** Mafalda Calheiros-Lobo, João P. N. Silva, Leonor Delgado, Bárbara Pinto, Luís Monteiro, Carlos Lopes, Patrícia M. A. Silva, Hassan Bousbaa

**Affiliations:** 1 UNIPRO—Oral Pathology and Rehabilitation Research Unit, University Institute of Health Sciences (IUCS), Cooperativa de Ensino Superior Politécnico e Universitário (CESPU), Rua Central de Gandra, 1317, 4585-116 Gandra, Portugal; mafalda.duarte@iucs.cespu.pt (M.C.-L.); joaosilva_06@hotmail.com (J.P.N.S.); mleonor.delgado@iucs.cespu.pt (L.D.); barbara.pinto@iucs.cespu.pt (B.P.); luis.monteiro@iucs.cespu.pt (L.M.); carlos.lopes@iucs.cespu.pt (C.L.); 2 Pathology Department, INNO Serviços Especializados em Veterinária, 4710-503 Braga, Portugal; 3 Department of Physiology and Biophysics, Institute of Biological Sciences, Federal University of Minas Gerais (UFMG), Av. Pres. Antônio Carlos, 6627, Belo Horizonte 31270-901, Brazil; 4 Medicine and Oral Surgery Department, University Institute of Health Sciences—CESPU (IUCS-CESPU), 4585-116 Gandra, Portugal; 5 Associate Laboratory i4HB, Institute for Health and Bioeconomy, University Institute of Health Sciences—CESPU, 4585-116 Gandra, Portugal; 6 UCIBIO—Applied Molecular Biosciences Unit, Translational Toxicology Research Laboratory, University Institute of Health Sciences (1H-TOXRUN, IUCS-CESPU), 4585-116 Gandra, Portugal

**Keywords:** MPS-1 inhibitor, aurora-B inhibitor, KSP inhibitor, cetuximab, oral cancer, antimitotics, combination treatment, antitumor activity

## Abstract

**Background/Objectives:** Head and neck cancer (HNC) is among the most common cancer types globally, with its incidence expected to increase significantly in the coming years. Oral squamous cell carcinoma (OSCC), the predominant subtype, exhibits significant heterogeneity and resistance to treatment. Current therapies, including surgery, radiation, and chemotherapy, often result in poor outcomes for advanced stages. Cetuximab, an EGFR inhibitor, is widely used but faces limitations. This study explores the combined inhibition of EGFR and mitotic proteins to enhance treatment efficacy. **Methods:** We analyzed the effects of co-treating OSCC cells with small molecules targeting MPS-1 (BAY1217389), Aurora-B (Barasertib), or KSP (Ispinesib), alongside Cetuximab. The rationale is based on targeting EGFR-mediated survival pathways and the mitotic checkpoint, addressing multiple cell cycle phases and reducing resistance. **Results:** Our findings indicate that inhibiting MPS-1, Aurora-B, or KSP enhances Cetuximab’s therapeutic potential, promoting increased cancer cell death. Additionally, we examined EGFR, MPS-1, Aurora-B, and KSP expression in OSCC patient samples, revealing their clinicopathologic significance. **Conclusions:** This combinatorial approach suggests a promising strategy to improve treatment outcomes in OSCC.

## Simple Summary

Head and neck cancer (HNC), especially oral squamous cell carcinoma (OSCC), is a common and increasingly prevalent cancer worldwide. OSCC is challenging to treat due to its aggressive nature and resistance to standard therapies like surgery, radiation, and chemotherapy, particularly in advanced stages. Cetuximab, a drug targeting EGFR (a protein that supports cancer cell growth), is often used but has limitations in effectiveness. This study explores a new approach by combining Cetuximab with drugs that target proteins involved in cell division, specifically MPS-1, Aurora-B, and KSP. These proteins help cancer cells progress through the cell cycle and are crucial for tumor survival. By blocking both EGFR and these cell division proteins, the study aimed to increase the effectiveness of Cetuximab in killing OSCC cells. Results showed that targeting MPS-1, Aurora-B, or KSP alongside EGFR led to more cancer cell death, suggesting that this combined approach could reduce treatment resistance. Analysis of patient samples confirmed that these proteins are significant in OSCC. This combined therapy strategy shows promise for improving outcomes in OSCC and potentially other head and neck cancers.

## 1. Introduction

Head and neck cancer squamous cell carcinoma (HNSCC) ranks as the sixth most common cancer subtype globally, with over 870,000 new cases and 325,000 deaths reported annually [1]. The incidence of HNSCC is increasing, with projections suggesting a 30% rise in cases by 2030. HNSCCs can be classified into two groups according to human papillomavirus (HPV) status: HPV-negative and HPV-positive. HPV-positive HNSCCs are more commonly associated with the oropharynx, hypopharynx, and larynx and have a better prognosis [2]. Oral squamous cell carcinoma (OSCC), the predominant histological subtype of HNSCC, accounts for approximately 90% of all cases. OSCC originates from the epithelial cells lining the mucosal surfaces of the oral cavity, oropharynx, larynx, or hypopharynx [3]. OSCC is characterized by significant heterogeneity, exhibiting a variety of genetic and epigenetic alterations associated with distinct risk factors [4]. These alterations confer several advantages to cancer cells, including the ability to proliferate independently of growth factors, resist apoptosis, and effectively breach extracellular matrix barriers, facilitating invasion into adjacent or distant tissues. These characteristics contribute to the aggressive nature of OSCC and its frequent resistance to treatment [5].

The treatment strategy for OSCC typically involves a multimodal approach, and surgery is the primary treatment for both early and advanced OSCC. It is often supplemented with radiotherapy (RT) and/or chemotherapy (CT) for patients with pathological adverse features [6]. The overall survival rate for patients with advanced stages of OSCC cell carcinoma remains low, and thus, new treatment options need to be explored [7]. One of the most widely used drugs for OSCC treatment is Cetuximab, a monoclonal antibody that targets the epidermal growth factor receptor (EGFR). The EGFR, a receptor tyrosine kinase (RTK) within the HER/ErbB family (which also includes HER2-4), is overexpressed in 80–90% of HNC cases and is associated with poor prognosis and treatment outcomes [8]. The EGFR signaling network is complex, involving numerous components and intersecting with multiple other pathways [9]. In human cancers, the activation of RTK signaling pathways is driven by various mechanisms, including ligand or receptor overexpression, aberrant ligand binding, and gene rearrangements. These processes enhance tumor cell migration, survival, and proliferation, contributing to the malignancy’s aggressive nature [10].

To counteract the development of aberrant cells, a network of quality control mechanisms, including checkpoints, operates throughout multiple phases of the cell cycle. Monopolar spindle 1 (MPS-1), alternatively known as threonine tyrosine kinase (TTK), serves as a vital component of the spindle assembly checkpoint (SAC). The SAC upholds the cell cycle at mitosis until all chromosomes have established stable bipolar attachments to the mitotic spindle and are aligned at the metaphase plate [11]. Deficient SAC activity leads to premature exit from mitosis, resulting in the generation of aneuploid cells due to chromosome mis-segregation. MPS-1 exhibits heightened expression levels across various cancer types, including breast cancer [12], hepatocellular carcinoma [13], pancreatic cancer [14], and gastric cancer [15], and its overexpression is mostly correlated with unfavorable patient prognosis. Notably, in breast cancer cell lines, downregulation of MPS-1 expression has been observed to diminish cell viability, highlighting its potential as a promising therapeutic target [16]. Importantly, Aurora-B, a member of the Aurora kinase family, including Aurora-A and Aurora-C, also plays a pivotal role in chromosome attachment and alignment, segregation, and cytokinesis. The inhibition of Aurora-B can induce polyploidy and subsequent cell death [17]. Overexpression of Aurora-B has been observed in metastatic and poorly differentiated OSCC, indicating its involvement in OSCC progression [18,19]. Kinesin spindle protein (KSP), also known as Eg5 or Kif11, is a member of the kinesin-5 family essential for the formation of bipolar mitotic spindles, the cross-linking of microtubules, and the proper chromosome alignment [20,21]. Its inhibition results in the formation of monopolar spindles, activation of the SAC, mitotic arrest, and subsequent cell death [22]. Overexpression of KSP is linked to poor prognoses in hepatocellular [23], breast [24] and laryngeal cancers [25].

Due to the critical role these proteins play in cell proliferation and their high expression levels in various types of cancer, which are linked with poor patient prognosis, several inhibitors have been developed. However, despite the promising results from preclinical trials, inhibitors of these proteins have shown disappointing outcomes as a monotherapy in clinical trials [26,27,28,29,30]. Several explanations were proposed for this lack of efficacy. One significant factor is that antimitotic agents act exclusively during mitosis, leading to low efficacy since only a small fraction of tumor cells undergo mitosis at any given time. Additionally, mitotic slippage, a phenomenon where cells exit mitosis without division, leading to aneuploidy and promoting cancer cell survival, has been identified as a major cause of resistance to antimitotic treatments [31].

The limitations of antimitotic agents as monotherapies highlight their reduced efficacy, which leads to cancer cell survival. Thus, alternative therapeutic strategies are needed. In this sense, we propose combining Cetuximab with antimitotic agents. The rationale for combining antimitotic inhibition with Cetuximab, an EGFR inhibitor, to enhance the death of oral cancer cells is based on the complementary roles these proteins play in cell proliferation and survival. By targeting both EGFR-mediated survival pathways and the mitotic checkpoint, this combination therapy aims to enhance the overall anti-cancer effect, leading to increased cancer cell death and improved treatment outcomes in oral cancer. Cetuximab primarily targets the G1 phase of the cell cycle, preventing cells from entering the S phase due to insufficient growth signals. In contrast, antimitotics target cells in the M phase. This combination ensures that cancer cells are targeted at multiple points in the cell cycle, reducing the likelihood of escape and resistance development and potentially leading to more effective and durable responses. Moreover, tumors often develop resistance to single-agent therapies through various mechanisms, such as compensatory signaling pathways or mutations.

The primary objective of this study was to comprehensively analyze the effects of co-treating oral cancer cells with small molecules targeting mitotic proteins, specifically an MPS-1 inhibitor (BAY1217389), an Aurora-B inhibitor (Barasertib), and a KSP inhibitor (Ispinesib), in combination with an EGFR inhibitor (Cetuximab). The EGFR, MPS-1, Aurora-B, and KSP expression patterns and the clinicopathologic significance in samples from OSCC patients were also meticulously analyzed.

## 2. Materials and Methods

### 2.1. Inhibitors

Inhibitors targeting EGFR (Cetuximab), MPS-1 (BAY1217389), Aurora-B (Barasertib), and KSP (Ispinesib) were obtained from MedChem Express (Shanghai, China) and reconstituted in sterile dimethyl sulfoxide (DMSO, Sigma-Aldrich Co., Ltd., St. Louis, MO, USA) to 10 mM or 5 mM stock concentrations. After reconstitution, aliquots were prepared from each inhibitor and promptly stored at −20 °C to mitigate the risk of compound degradation from repeated freeze–thaw cycles. Fresh working solutions were prepared in a culture medium on the day of the experiment to achieve the desired inhibitor concentrations.

### 2.2. Cell Lines and Culture Conditions

Two human oral cancer cell lines were used in this study: SCC-09 (Tongue Squamous Cell Carcinoma; The Global Bioresource Center-ATCC^®^ CRL-1628) and SCC-25 (Tongue Squamous Cell Carcinoma; The Global Bioresource Center-ATCC^®^ CRL-1629). The cells were maintained in a DMEM-F12 culture medium (Roswell Park Memorial Institute, Biochrom, Buffalo, NY, USA), supplemented with 10% fetal bovine serum (FBS, Biochrom, Berlin, Germany) and 40 ng/mL of hydrocortisone (Sigma-Aldrich). According to the manufacturer’s instructions, human oral keratinocytes (HOK, ScienCell Research Laboratories, Carlsbad, CA, USA) were maintained in a specific HOK medium (Innoprot, Biscaia, Spain). All cell lines were cultured at 37 °C in a humidified atmosphere containing 5% CO_2_ (Hera Cell, Heraeus, Hanau, Germany).

### 2.3. RNA Extraction, cDNA Synthesis, and Quantitative Real-Time PCR

Extraction of total RNA extraction and the subsequent synthesis of cDNA were carried out as previously described [31]. After, the iQ^TM^ SYBR Green Supermix Kit (Bio-Rad Laboratories, Inc., Hercules, CA, USA) was used for DNA amplification on an iQ Thermal Cycler (Bio-Rad), with the following protocol: initial denaturation step at 95.0 °C for 3 min, followed by 40 cycles of denaturation at 94.0 °C for 20 s, annealing at 62.0 °C for 30 s, and extension at 72.0 °C for 30 s. The melt curve analysis encompassed temperatures ranging from 65.0 to 95.0 °C, incremented by 0.5 °C for 5 s. The sequences of the primers for the amplification of MPS-1 were as follows: forward 5′-CCGAGATTTGGTTGTGCCTGGA-3′ and reverse 5′-CATCTGACACCAGAGGTTCCTTG-3′. For the amplification of EGFR, they were as follows: forward 5′-AGGCACGAGTAACAAGCTCAC-3′ and reverse 5′-ATGAGGACATAACCAGCCACC-3′. For the amplification of actin, they were as follows: forward 5′-AATCTGGCACCACACCTTCTA-3′ and reverse 5′-ATAGCACAGCCTGGATAGCAA-3′. For the amplification of GAPDH, they were as follows: forward 5′-GTCTCCTCTGACTTCAACAGCG-3′ and reserve 5′-ACCACCCTGTTGCTGTAGCCAA-3′. Actin and GAPDH were used as a reference control to normalize the data. Each independent experiment (n = 3) was performed in triplicate, and the data was acquired using the CFX Manager^TM^ Software (version 1.0, Bio-Rad). The relative quantification was calculated using the ∆∆CT method.

### 2.4. Protein Extracts and Western Blotting

The protein extracts retrieved from SCC-09, SCC-25, and HOK-cell pellets were suspended in lysis buffer composed of 50 mM Tris (pH 7.5), 150 mM NaCl, 1 mM EDTA, 1% Triton-100, and a protease inhibitor cocktail (Sigma-Aldrich). Following the manufacturer’s guidelines, protein quantification was performed using the BCATM Protein Assay Kit (Pierce Biotechnology, Rockford, IL, USA). For MPS-1 and EGFR detection, 15 and 20 μg of protein lysate, respectively, were reconstituted in SDS-sample buffer (consisting of 375 mM Tris pH 6.8, 12% SDS, 60% Glycerol, 0.12% Bromophenol Blue, and 600 nM DTT) and boiled for 3 min.

Then, a 7.5% gradient gel (Bio-Rad) was used for protein separation. After SDS-PAGE, proteins were transferred onto nitrocellulose membranes (Amersham, UK) using the Trans-Blot Turbo Transfer System (Bio-Rad). Following the transfer, the membranes were blocked in a 5% non-fat dried milk (*w*/*v*) dissolved in TBST buffer (comprising 50 mM Tris pH 7.5, 150 mM NaCl, and 0.05% Tween-20) and incubated overnight at 4 °C with primary antibodies diluted in TBST. The primary antibodies used included mouse anti-α-tubulin (1:5000, 1:5000, T568 Clone B-5-1-2, Sigma-Aldrich), mouse anti-EGFR (1:250, HPA044700, Sigma-Aldrich), and mouse anti-MPS-1 (1:1000, (N1): sc-56968, Santa Cruz Biotechnology, Heidelberg, Germany). After three washes with TBST, the membranes were incubated with horseradish-peroxidase-conjugated secondary antibodies (1:1500 for anti-mouse and 1:1000 for anti-rabbit) for 1 h at room temperature.

A ChemiDOc system (Bio-Rad) was then used to visualize protein bands after exposure to the Enhanced Chemiluminescence (ECL) method. Image Lab 6.1v software or Image J (version 1.47, Rasband, W.S., ImageJ, U. S. National Institutes of Health, Bethesda, MD, USA)was used to quantify protein signal intensity with normalization against α-tubulin expression levels.

### 2.5. MTT Viability Assay

The cell viability was determined by tetrazolium salt 3-(4, 5-dimethylthiazol-2-yl)-2, 5-diphenyltetrazolium bromide (MTT) assay. In summary, SCC-09 and SCC-25 cells were seeded in 96-well plates at a density of 0.1 × 10^6^ and 0.05 × 10^6^ cells/mL, respectively. After 24 h, the culture medium was replaced with fresh medium containing 2-fold serial dilutions of Cetuximab ranging from 0 to 120 nM in combination with Barasertib (SCC-09 and SCC-25) and from 0 to 240 nM in combination with BAY1217389 and Ispinesib (SCC-09 and SCC-25), with BAY1217389 ranging from 0 to 6400 nM, Barasertib ranging from 0 to 16,000 nM (SCC-25) and from 0 to 64,000 nM (SCC-09), or Ispinesib ranging from 0 to 30 nM (SCC-25) and from 0 to 60 nM (SCC-09). After a 48 h incubation at 37 °C and 5% CO_2_, the medium was removed and replaced with a combination of 200 μL of DMEM F12 only and 20 μL of tetrazolium salt MTT (5 mg/mL PBS). Afterward, the plates were placed in an incubator at 37 °C for 2–4 h, allowing formazan crystals to form. Then, the medium was extracted, and the formazan crystals were resuspended in 100 μL of DMSO. Next, the plates were placed in a microplate reader (Biotek Synergy 2, Winooski, VT, USA) coupled with Gen5 software (version 1.07.5, Biotek, Winooski, VT, USA) to measure the absorbance using a wavelength of 570 nm.

Cell viability was assessed as a percentage relative to the control group and presented as the mean ± standard deviation from three independent experiments, each performed in triplicate. IC_50_ values, representing the mean 50% inhibitory concentration, were determined using GraphPad Prism version 8 (GraphPad software Inc., San Diego, CA, USA). The combination treatment effects were analyzed using a dual-drug crosswise concentration matrix for each combination, applying the specified concentration ranges. The results were then analyzed using Combenefit Software (version 2.021, Cancer Research UK Cambridge Institute, Cambridge, UK).

### 2.6. Apoptosis Detection by Flow Cytometry

The assessment of apoptotic cell death was performed using the Annexin V-FITC Apoptosis Detection Kit (eBioscience, Vienna, Austria) in accordance with the manufacturer’s protocol. Initially, cells were seeded at a 0.1 × 10^6^ cells/mL density in 6-well plates. After 24 h of incubation, the cells were treated with EGFR, MPS-1, Aurora-B, and KSP inhibitors either individually or in combination, using concentrations corresponding to their respective synergistic points (30 nM of Cetuximab with 40 nM of BAY1217389, 15 nM of Cetuximab with 1000 nM of Barasertib, and 240 nM of Cetuximab with 1.875 nM of Ispinesib). Following a 24 h treatment with Ispinesib and Cetuximab and a 48 h incubation period for the other combinations, adherent and floating cells were harvested and pelleted by centrifugation at 1000 rpm for 5 min. The pelleted cells were then suspended in binding buffer 1×, followed by the addition of Annexin V-FITC, and incubated for 10 min in darkness. After washing, the cells were resuspended once again in binding buffer 1×, followed by the addition of Propidium iodide (PI) at a concentration of 20 μg/mL. A BD Accuri™ C6 Plus Flow cytometer (BD Biosciences, San Jose, CA, USA) was used for the fluorescence analysis. The acquired data was then processed using BD Accuri™ C6 Plus software, version 1.0.27.1.

For data analysis, 20,000 events were recorded per sample.

### 2.7. Colony Formation Assay

A total of 1000 SCC-25 cells were seeded into six-well plates and allowed to adhere for 24 h. Subsequently, the cells were exposed to drug treatments, administered either as monotherapies or in combination. Control groups consisted of untreated cells and DMSO-treated ones. The cells were incubated for 48 h with the respective treatments and then were rinsed twice with PBS. After the drug-free DMEM medium was added, the cells were maintained for 7 days in these conditions. Colonies fixation was performed by the addition of 100% methanol at −20 °C for 25 min, and then they were stained with a 0.05% (*w*/*v*) solution of crystal violet (Merck, Rahway, NJ, USA) in distilled water for 20 min. Three independent experiments were used to obtain the colony counts. The ratio of the number of colonies to the number of cells seeded in the control group was used to calculate the plating efficiency (PE), expressed as a percentage. The survival fraction for each condition was calculated by dividing the number of colonies by the number of cells seeded and then multiplying by the reciprocal of the PE.

### 2.8. Immunohistochemistry

#### 2.8.1. Patients and Tissue Specimens

This study was approved by the institutional ethical board of the Hospital de Santo António (HSA), Centro Hospitalar do Porto, Portugal (Investigation, Formation, and Teaching Department—DEFI; 024/CES/03). Written informed consent was obtained from all participants. The research was conducted following the Declaration of Helsinki. Tissue samples from primary Oral Squamous Cell Carcinoma (OSCC) (ICD 10: C00-06) were retrospectively collected from 2000 to 2006 at the abovementioned hospital. Clinical characteristics of the OSCC patients are summarized in Supporting Information Table S1.

#### 2.8.2. Processing and Evaluation

Immunohistochemistry on tissue microarray (TMA) sections was performed using the Novolink Polymer Detection System (Novocastra, Leica Biosystems Newcastle Ltd., Newcastle Upon Tyne, UK), following the protocol outlined by Monteiro et al. [32]. For TMA construction, two representative regions of OSCC from the invasive fronts of the tumors were chosen from hematoxylin and eosin-stained sections, deliberately excluding areas of keratin and necrosis. From each selected specimen, two tissue cores, each 2 mm in diameter, were extracted and embedded into a paraffin TMA block using the TMA Builder (Histopathology Ltd., Pécs, Hungary). Technical controls were represented by non-neoplastic tissue cores [33]. The primary antibodies utilized were mouse anti-human EGFR (1:500, HPA044700, Sigma-Aldrich), mouse anti-human MPS-1 (1:100, clone EPR5319(2), ab133699, Abcam, Cambridge, UK), rabbit anti-KSP (1:300, Abcam), and rabbit anti-Aurora-B (1:50, Sigma-Aldrich). Normal colon tissue was used as a positive control, while negative control sections were incubated without the primary antibody. Staining was measured semi-quantitatively independently by two authors blinded to clinicopathological data. For EGFR, we consider negative cases with a labeling index of <10% of tumor cells. We categorize cases with ≥10% of tumor cells and weak intensity as a score of 1+, moderate intensity score of 2+, and strong intensity as 3+. For MPS-1, Aurora-B and KSP intensity scores were evaluated using a scale of 0 (negative), 1 (weak), 2 (moderate), and 3 (strong) intensity. Discordant cases were reviewed under a multihead microscope to reach consensus; unresolved discordant cases were excluded. For each patient, the highest score from the three cores was used in analyses if scores differed. Cutoffs were determined according to ROC curves. For EGFR, Aurora-B, and KSP, staining intensity was classified as low for scores ≤ 2 and high for scores 3. For MPS-1, staining intensity was classified as low for scores ≤ 1 and high for scores 2 and 3 [34].

### 2.9. Image Acquisition and Processing

Phase-contrast microscopy images were acquired using a Nikon TE 2000-U microscope (Nikon, Amsterdam, The Netherlands) with a 20× objective lens. The microscope was interfaced with a DXM1200F digital camera operated through Nikon ACT-1 software version 2.63 (Melville, NY, USA). Post-imaging processing and analysis were performed utilizing ImageJ software.

### 2.10. Bioinformatic Analysis

The UALCAN database (http://ualcan.path.uab.edu/ (accessed on 11 September 2024)) was used to analyze the expression of EGFR1, MPS-1, Aurora-B, and KSP in HNSCC and examine their association with the clinicopathologic characteristics of HNSCC patients. The screening parameters were set as follows: “Gene: EGFR, TTK (MPS-1), AURKB (Aurora-B), or Kif11 (KSP)” and “Cancer Type: Head and Neck Squamous Cell Carcinoma”. The analysis type was defined based on the target variable, such as “HNSCC vs. Normal Analysis”. Transcriptomic data were sourced from The Cancer Genome Atlas (TCGA) via UALCAN, while proteomic data were retrieved from the Clinical Proteomic Tumor Analysis Consortium (CPTAC). Transcriptomic results were expressed as transcripts per million (TPM), and proteomic results were provided as Z-values, representing standard deviations from the median across HNSCC samples. Pearson correlation analysis was performed within UALCAN to calculate the Pearson correlation coefficient. For overall survival (OS) and disease-free survival (DFS) analysis, the GEPIA web tool (http://gepia.cancer-pku.cn/ (accessed on 11 September 2024)) was used, with the median as the group cutoff and a 95% confidence interval for statistical reliability. Data are presented as means ± standard deviation (SD), with statistical significance (*p*-values) provided by UALCAN or GEPIA.

### 2.11. Statistical Analysis

All experiments were performed in triplicate and repeated in at least three independent trials. Data are presented as mean values with standard deviation (SD). Statistical analyses were conducted using GraphPad Prism Software Inc. version 8, applying either an unpaired *t*-test or two-way analysis of variance (ANOVA) followed by Tukey’s multiple comparisons test. Statistical significance was denoted as * *p* < 0.05, ** *p* < 0.01, *** *p* < 0.001, and **** *p* < 0.0001. Univariate survival analysis was carried out using Kaplan–Meier curves and the log-rank test, while the Cox regression model was employed to assess the independent significance variables identified in univariate analysis.

## 3. Results

### 3.1. EGFR, MPS-1, Aurora-B, and KSP Are Overexpressed in HNSCC and Are Correlated with Clinical Features

**Figure 1:**
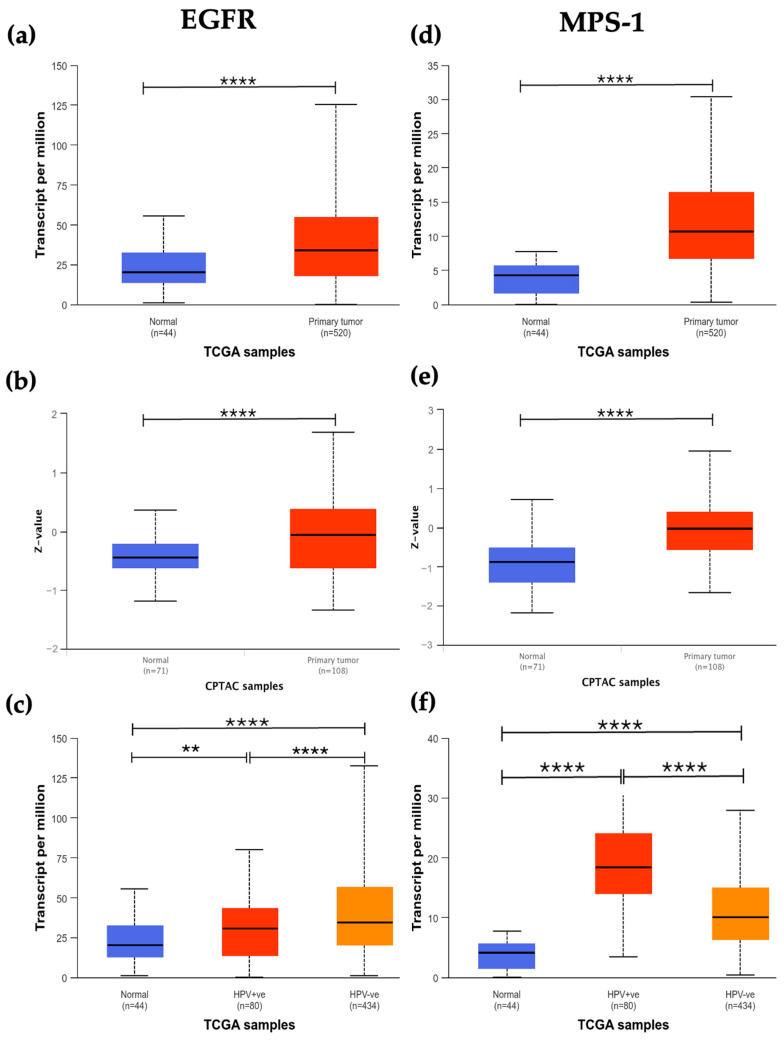

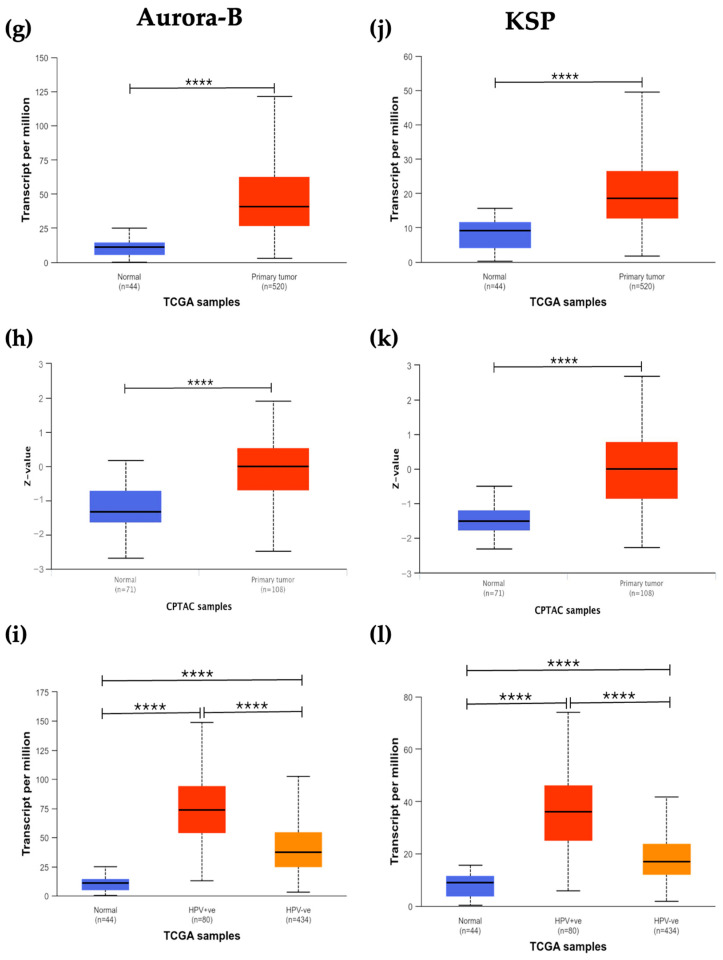
mRNA and protein expression of EGFR (**a**,**b**), MPS-1 (**d**,**e**), Aurora-B (**g**,**h**) and KSP (**j**,**k**) in HNSCC patients. mRNA expression of EGFR (**c**), MPS-1 (**f**), Aurora-B (**i**) and KSP (**l**) is increased in both HPV positive and negative patients. The significance levels were as follows: ** *p* < 0.01, **** *p* < 0.0001. Data were retrieved from UALCAN (http://ualcan.path.uab.edu/) on 11 September 2024. Abbreviations: CPTAC—Clinical Proteomic Tumor Analysis Consortium; TCGA—The Cancer Genome Atlas; HNSCC—Head and Neck Squamous Cell Carcinoma; EGFR—Epidermal Growth Factor Receptor; mRNA—messenger ribonucleic acid; UALCAN—University of Alabama at Birmingham Cancer data analysis Portal.

To investigate the expression and assess the potential as biomarkers and treatment targets of EGFR, MPS-1, Aurora-B, and KSP in HNSCC patients, we explored both the UALCAN and GEPIA databases. The analysis revealed that all four proteins are overexpressed at both the mRNA and protein levels compared to normal tissue samples (Figure 1a,b,d,e,g,h,j,k). Furthermore, when examining HPV status, we observed elevated mRNA levels of EGFR, MPS-1, Aurora-B, and KSP in both HPV-negative and HPV-positive samples (Figure 1c,f,i,l). Notably, HPV-positive samples exhibited higher mRNA expression levels for MPS-1, Aurora-B, and KSP, with EGFR showing a less pronounced but still significant increase.

**Figure 2:**
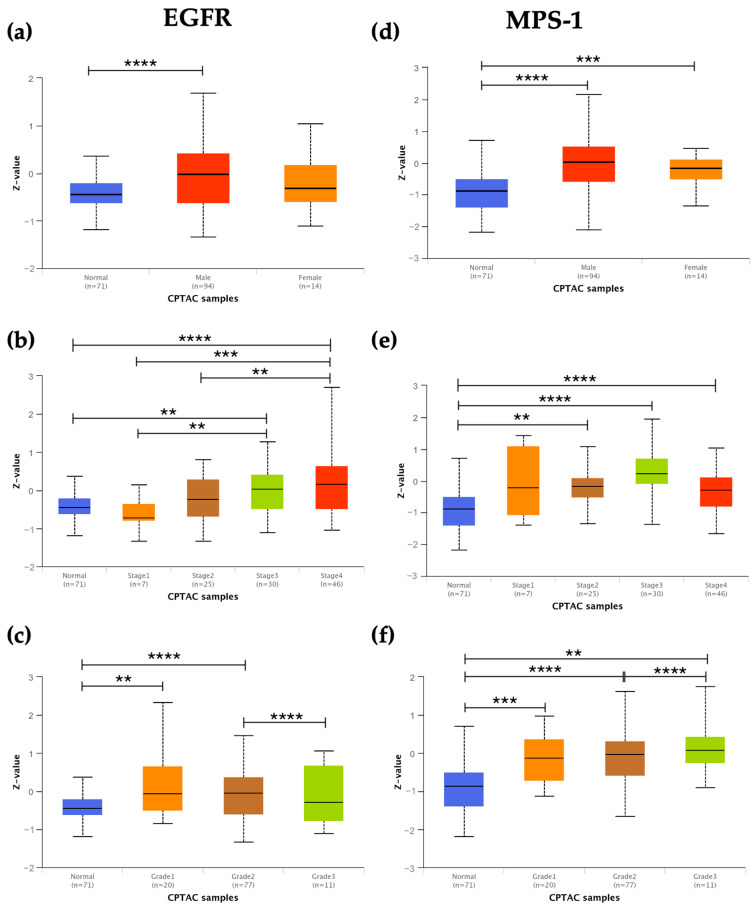

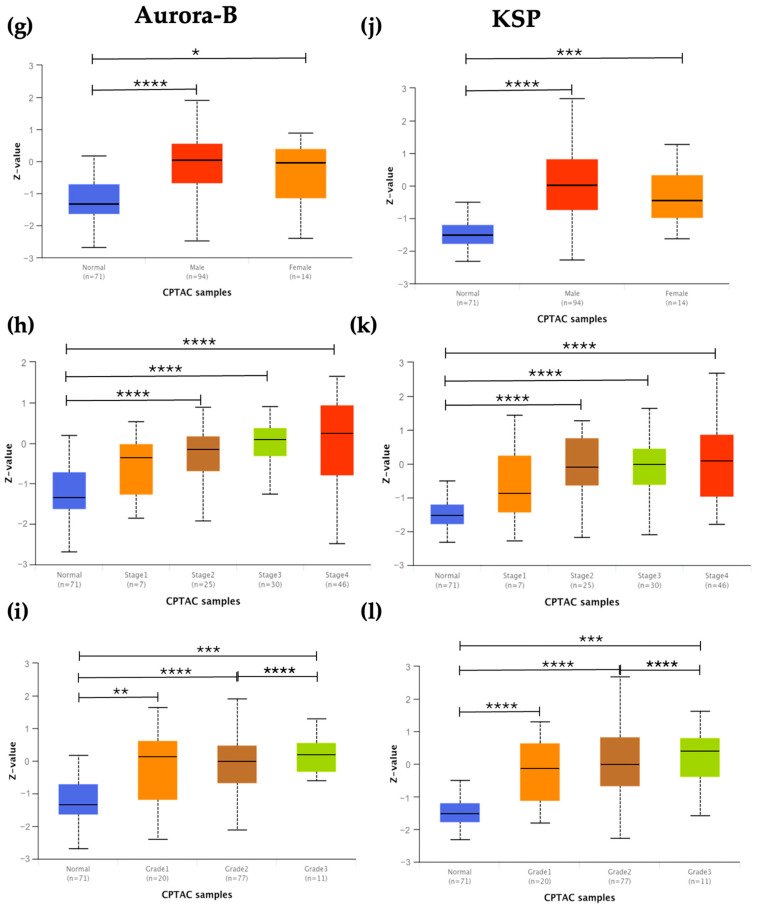
Association of HNSCC patients clinicopathological features with expression levels of EGFR, MPS-1, Aurora-B, and KSP. Correlation of EGFR, MPS-1, Aurora-B, and KSP expression with gender (**a**,**d**,**g**,**j**) tumor stage (**b**,**e**,**h**,**k**) and grade (**c**,**f**,**i**,**l**). The significance levels were as follows: * *p* < 0.05, ** *p* < 0.01, *** *p* < 0.001 and **** *p* < 0.0001. Data were retrieved from UALCAN (http://ualcan.path.uab.edu/) on 11 September 2024. Abbreviations: HNSCC—Head and Neck Squamous Cell Carcinoma; EGFR—Epidermal Growth Factor Receptor; KSP—Kinesin Spindle Protein; UALCAN—University of Alabama at Birmingham Cancer data analysis Portal.

After we explored the correlation of EGFR, MPS-1, Aurora-B, and KSP expression and clinicopathological features of patients with HNSCC, we observed that EGFR was overexpressed exclusively in male patients (Figure 2a), whereas MPS-1, Aurora-B, and KSP were overexpressed in both male and female patients compared to normal samples (Figure 2d,g,j). Additionally, even though not statistically significant, male patients demonstrated higher levels of protein expression than female patients.

Moreover, overexpression of MPS-1, Aurora-B, and KSP was observed across all tumor stages and grades, except for patients with stage 1 tumors, compared to normal samples (Figure 2e,f,h,i,k,l). In relation to tumor stages, EGFR showed overexpression in stages 3 and 4 compared to normal samples, with statistically significant differences noted between stage 1 and stages 3–4, as well as between stage 2 and stage 4 (Figure 2b). Regarding tumor grades, EGFR was overexpressed in grades 1 and 2, with a statistically significant difference observed between grade 2 and grade 3 (Figure 2c).

**Figure 3:**
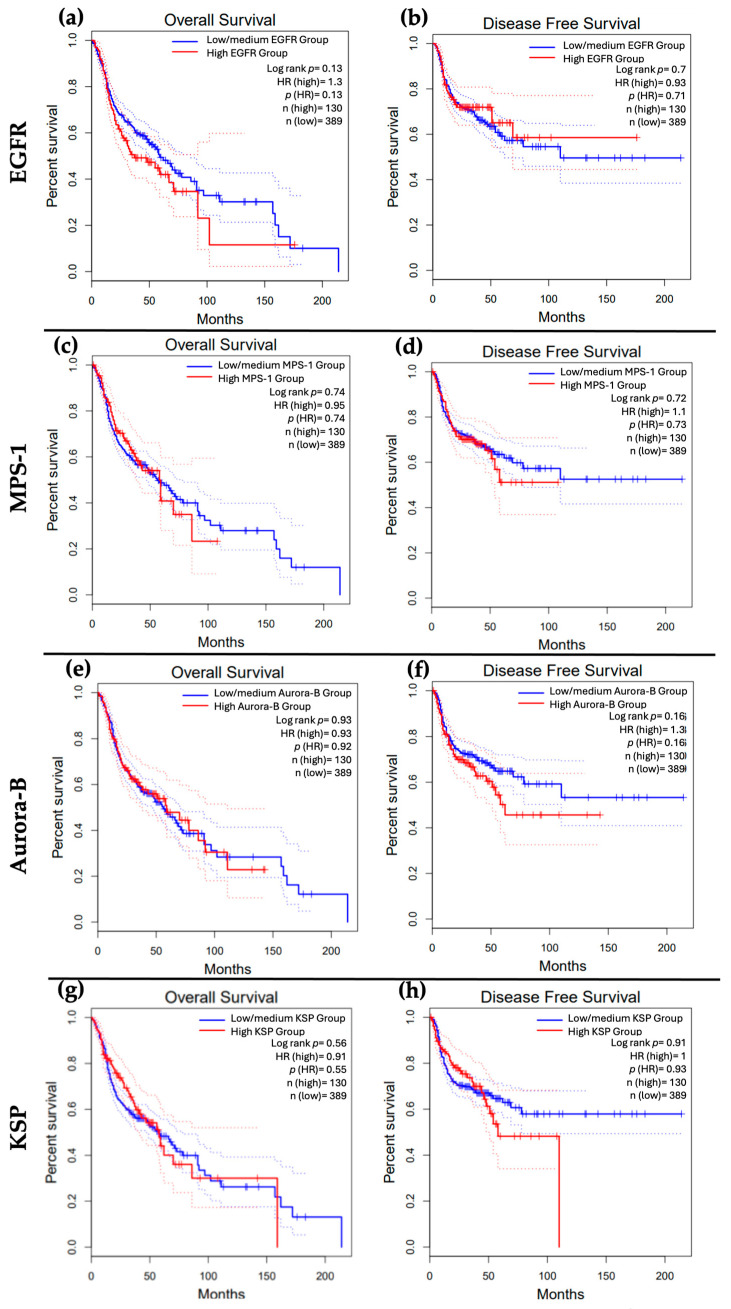
Effect of EGFR, MPS-1, Aurora-B, and expression on HNSCC patients’ overall survival (OS) (**a**,**c**,**e**,**g**) and disease-free survival (DFS) (**b**,**d**,**f**,**h**). Data were retrieved from the GEPIA (http://gepia.cancer-pku.cn/) database on 11 September 2024. Abbreviations: AURKB—Aurora-B; EGFR—GEPIA—Gene Expression Profiling Interactive Analysis; KSP—Kinesin Spindle Protein; MPS-1—Monopolar spindle 1.

We subsequently explored the impact of overexpression of EGFR, MPS-1, Aurora-B, and KSP on the survivability of patients with HNSCC. Our analysis indicated that high expression levels of EGFR were associated with a trend toward worse overall survival (OS), while the opposite was observed for disease-free survival (DFS) (Figure 3a,b). Furthermore, MPS-1 overexpression appeared to correlate with poorer outcomes for both OS and DFS (Figure 3c,d). Although there was a trend suggesting that patients with high Aurora-B expression had lower DFS, this finding was not statistically significant. Additionally, the OS for patients with low and high Aurora-B expression was similar (Figure 3e,f). Notably, while not statistically significant, patients exhibiting high KSP expression demonstrated a tendency toward worse OS and DFS outcomes (Figure 3g,h).

These results seem to suggest that the proteins explored in this study could have potential therapeutic targets in HNSCC, even though no clear association between their overexpression and survivability was observed. Nonetheless, their overexpression showed a tendency towards a worse OS; however, further research is warranted to establish statistical significance and to elucidate the precise nature of these associations.

### 3.2. EGFR, MPS-1, Aurora-B, and KSP Are Overexpressed in OSCC Patient Tissues

After the detailed UALCAN and GEPIA analysis, we proceeded to analyze samples from 30 patients with oral cancer to investigate their immunohistochemistry features and possible correlations with survivability. Firstly, EGFR, MPS-1, Aurora-B, and KSP staining intensity and the extent of Aurora-B and KSP were assessed for potential associations with the clinicopathological characteristics of OSCC patients. These features included gender, age, tumor location, stage, treatment modality, grade, margin status, vascular invasion, perineural permeation, lymphatic invasion, and muscular invasion. While no significant correlation was identified between protein expression and clinicopathological characteristics, a trend emerged that may become clinically significant with an increased sample size (N). As previously mentioned, only staining intensity was analyzed for EGFR and MPS-1 due to uniformly high-extent values across all cases (Table 1). In contrast, both staining intensity and extent were evaluated for Aurora-B and KSP (Table 2 and Table 3).

**Figure 4:**
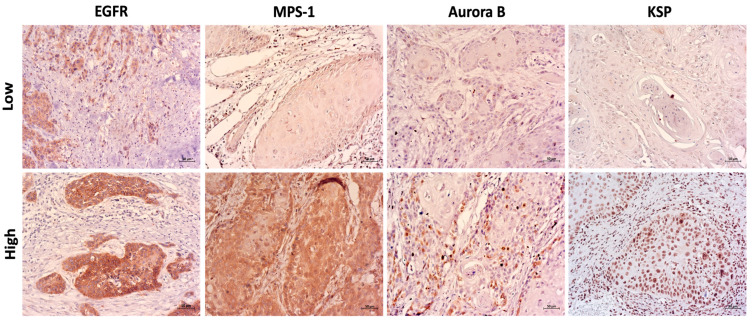
Immunohistochemical analysis of EGFR, MPS-1, Aurora-B, and KSP expression and localization in oral squamous cell carcinoma. Comparison between squamous cell carcinoma samples showing representative images in upper line correspondent to EGFR (75–100% and moderate intensity), MPS-1 (75–100% and weak intensity), Aurora-B (0–9% and weak intensity), and KSP (0–9% and weak intensity) low expression score. While the images in the lower line correspondent to EGFR (75–100% and strong intensity), MPS-1 (75–100% and strong intensity), Aurora-B (10–24% and strong intensity), and KSP (75–100% and strong intensity) high expression score. Cutoffs for the expression score were determined by ROC curves analysis. For EGFR, Aurora-B, and KSP, the staining intensity was classified as low for scores ≤ 2 and high for scores ≥ 3. While for MPS1, low intensity was considered for scores ≤ 1 and strong intensity for scores ≥ 2. Images at 200× magnification. Scale bar = 50 μm.

Immunohistochemical analysis was performed to assess the localization and expression of EGFR, MPS-1, Aurora-B, and KSP in paraffin-embedded OSCC samples (Figure 4). EGFR expression was observed in all 30 (100%) OSCC tissue microarrays, primarily localized on the membrane of the tumor cells. The expression ranged from 50 to 74% in 3 cases (10%) and 75 to 100% in 27 cases (90%). For data analysis, EGFR staining intensity was categorized into two groups: low intensity in 18 cases (62%) and high intensity in 11 cases (38%). MPS-1 expression was found in 25 cases, predominantly localized in the cytoplasm of the tumor cells, with expression levels classified as 50–74% in 1 case (4%) and 75–100% in 24 cases (96%). For data analysis, MPS-1 staining intensity was divided into two groups: negative to medium intensity (0, 1+) in 5 cases (20%) and moderate to high intensity (2+, 3+) in 20 cases (80%). Due to the high expression levels in all cases, only intensity values were considered for those two biomarkers. Aurora-B expression was found in 20 cases, localized to the nucleus of tumor cells, and classified as 0–9% in 11 cases (55%), 10–24% in 5 cases (25%), 25–49% in 2 cases (10%), and 50–74% in 2 cases (10%). For data analysis, Aurora-B expression was grouped into ≤9% expression in 11 cases (55%) and ≥10% expression in 9 cases (45%). Intensity staining followed the same categorization. KSP was found in 20 cases, and it was also localized to the nucleus of tumor cells. Expression was categorized as 0-9% in 4 cases (20%), 10–24% in 2 cases (10%), 50–74% in 4 cases (20%), and 75–100% in 10 cases (50%). For data analysis, KSP expression was grouped into ≤9% expression in 4 cases (20%) and ≥10% expression in 16 cases (80%). Staining intensity was recorded into two groups: negative to moderate intensity (0, 1+, 2+) with 16 cases (80%) and strong intensity (3+) with 4 cases (20%). A statistically significant correlation was observed between MPS-1 and KSP expression (*p* = 0.753 **), with staining intensity being directly proportional.

**Figure 5:**
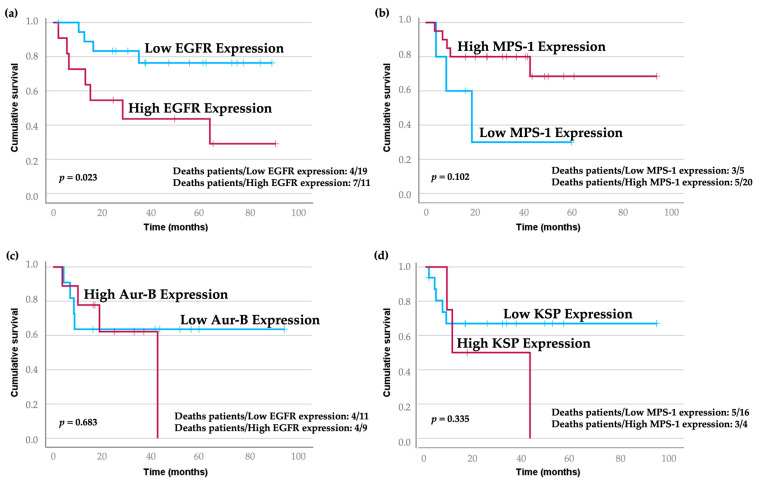
Kaplan–Meier curves illustrating overall patient survival based on expression levels of EGFR (**a**), MPS-1 (**b**), Aurora-B (**c**), and KSP (**d**). Blue lines correspond to cases with low expression, while red lines represent cases with high expression. Notably, higher EGFR staining intensity is significantly associated with reduced cancer-specific survival. Univariate analysis showed that patients with the highest EGFR expression had shorter survival times compared to those with lower expression levels (*p* = 0.023).

EGFR, MPS-1, Aurora-B, and KSP were also assessed for association with patient prognosis. The follow-up time for the 30 patients was 36 months. EGFR staining intensity is significantly associated with cancer-specific survival. Kaplan–Meier curves with univariate analyses showed that patients with the highest intensity of EGFR had reduced survival times compared to those with lower expression levels (*p* = 0.023). The stage of the tumor is also significantly associated with cancer-specific survival (*p* = 0.020), as is the treatment modality (*p* = 0.027) (Table 4 and Figure 5). No other variables were related to overall survival. These data highlight the significant prognostic value of tumor stage, treatment modality, and EGFR staining intensity in OSCC patients.

The variables evaluated with Kaplan–Meier curves, which demonstrated significant results in the Log-rank test, were subsequently incorporated into a multivariate analysis. This analysis showed that strong EGFR staining intensity is an independent prognostic factor associated with a significantly increased risk of reduced survival (HR = 4.745, *p* = 0.029) compared to individuals with low or moderate EGFR intensity. This finding suggests that high EGFR expression serves as a strong prognostic indicator in OSCC, consistent with previous studies linking EGFR overexpression to poor prognosis in various cancers, including OSCC. Furthermore, the type of treatment and tumor stage did not show significant associations with survival in this analysis. The lack of significant results for treatment modality could reflect the heterogeneity of grouped treatments or an insufficient sample size to detect a difference. Similarly, while advanced-stage disease typically correlates with poorer prognosis, the current sample may lack sufficient statistical power to confirm this relationship (Table 5).

In summary, we did not observe any correlation between the high expression levels of the proteins examined and the clinicopathological characteristics of OSCC patient samples, likely due to the limited sample size (low N). Notably, only high expression of EGFR exhibited statistical significance in relation to worse overall survival. Neither patient stage nor treatment modality demonstrated a significant correlation with patient survival. Furthermore, the multivariate analysis for the variables that showed significant results indicated that only EGFR intensity retained independent significance, highlighting an increased risk for a worse prognosis.

### 3.3. EGFR, MPS-1, Aurora-B, and KSP Are Overexpressed in Oral Cancer Cells

EGFR is a transmembrane glycoprotein that belongs to the RTK family, and its primary function is to regulate cellular processes such as growth, proliferation, differentiation, and survival in various cell types, including epithelial, glial, and neuronal cells [35]. In HNC, EGFR is overexpressed in 80–90% of cases and is associated with poor prognosis and unfavorable treatment outcomes [4]. MPS-1 is a critical regulator of chromosome alignment during metaphase, ensuring proper kinetochore-microtubule attachments to prevent aneuploidy [36]. Inhibitors of MPS-1 circumvent the SAC, inducing premature mitotic exit that results in extensive chromosome mis-segregation and, ultimately, cell death, thereby acting as potent mitotic drivers [37]. Aurora-B, a member of the Aurora kinase family, is responsible for monitoring and correcting improper attachments of microtubules to kinetochores, as well as regulating the dissociation of cohesin, a protein essential for maintaining cohesion between sister chromatids during mitosis [38]. KSP plays a crucial role in establishing spindle bipolarity and ensuring the proper separation of spindle poles; inhibition of KSP leads to the collapse of mitotic spindles and the formation of mono-aster [39].

**Figure 6:**
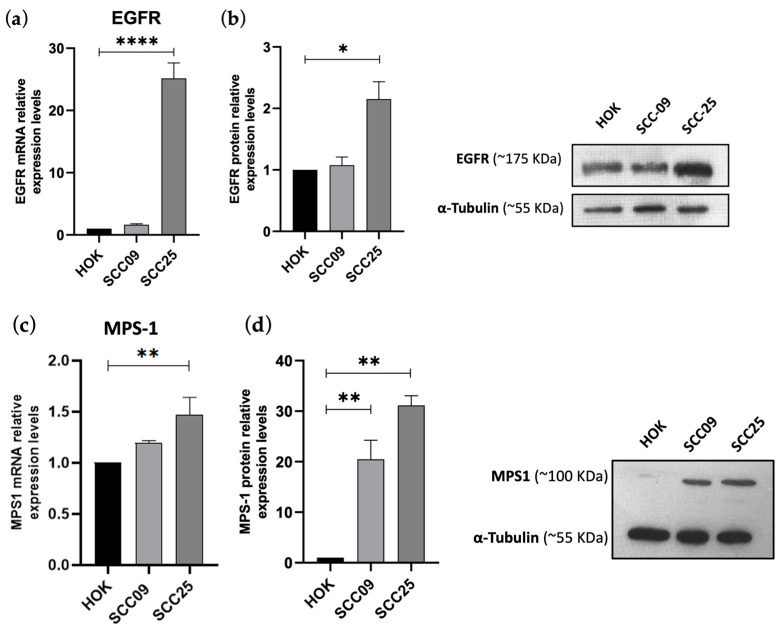
EGFR and MPS-1 are overexpressed in oral cancer cell lines. Relative expression of EGFR (**a**) and MPS-1 (**c**) mRNA as determined by qRT-PCR in SCC-09 and SCC-25 tumor cell lines, comparatively to non-tumor HOK. Representative Western Blots showing differential expression at protein levels of EGFR (**b**) and MPS-1 (**d**). α-tubulin was used as a loading control. The significance levels were as follows: * *p* < 0.05, ** *p* < 0.01 and **** *p* < 0.0001. Original western blots are presented in File S1.

To explore the therapeutic potential of targeting these proteins, we first assessed their expression at both the mRNA and protein levels in two oral cancer cell lines (SCC-09 and SCC-25) using qRT-PCR and Western blot analysis, respectively (Figure 6). Our results indicated that both EGFR and MPS-1 mRNA levels were significantly overexpressed in the SCC-25 cell line compared to the non-tumor cell line HOK (25-fold increase and 1.5-fold increase for EGFR and MPS-1, respectively) (Figure 6a,b). Regarding protein expression, we observed elevated levels of EGFR in the SCC-25 cell line, while MPS-1 was overexpressed in both cell lines (Figure 6c,d).

Our group previously analyzed and reported the expression of Aurora-B and KSP using the same cell lines, demonstrating that both proteins were overexpressed at the mRNA and protein levels [40].

Globally, the overexpression of EGFR, MPS-1, Aurora-B, and KSP in oral cancer cell lines highlights the relevance of their targeting to potentiate current oral cancer treatments.

### 3.4. Co-Treatment of Cetuximab with MPS-1, Aurora-B, or KSP Inhibitors Showed Synergistic Effects in Oral Cancer Cells

We then evaluated the cytotoxic effects of MPS-1, Aurora-B, and KSP inhibitors in combination with the EGFR inhibitor Cetuximab on oral cancer cells.

Using the MTT assay, we determined the IC_50_ of BAY1217389 and Cetuximab in both SCC-09 and SCC-25 cell lines and evaluated the cytotoxic effects of BAY1217389, Barasertib, Ispinesib, and Cetuximab, both individually and in combination (Table 6). The IC_50_ of Cetuximab could not be established, even at a concentration of 800 nM; therefore, we employed lower concentrations in the MTT assay to identify potential synergistic points with reduced drug concentrations. Nonetheless, BAY1217389 exhibited comparable IC_50_ for both cell lines (402.95 ± 4.31 nM vs. 540.6 ± 2.12 nM for SCC-25 and SCC-09, respectively). The IC_50_ values of Barasertib and Ispinesib were previously determined, and as indicated in Table 1, the SCC-25 cell line demonstrated greater sensibility to these drugs compared to the SCC-09 cell line.

**Figure 7:**
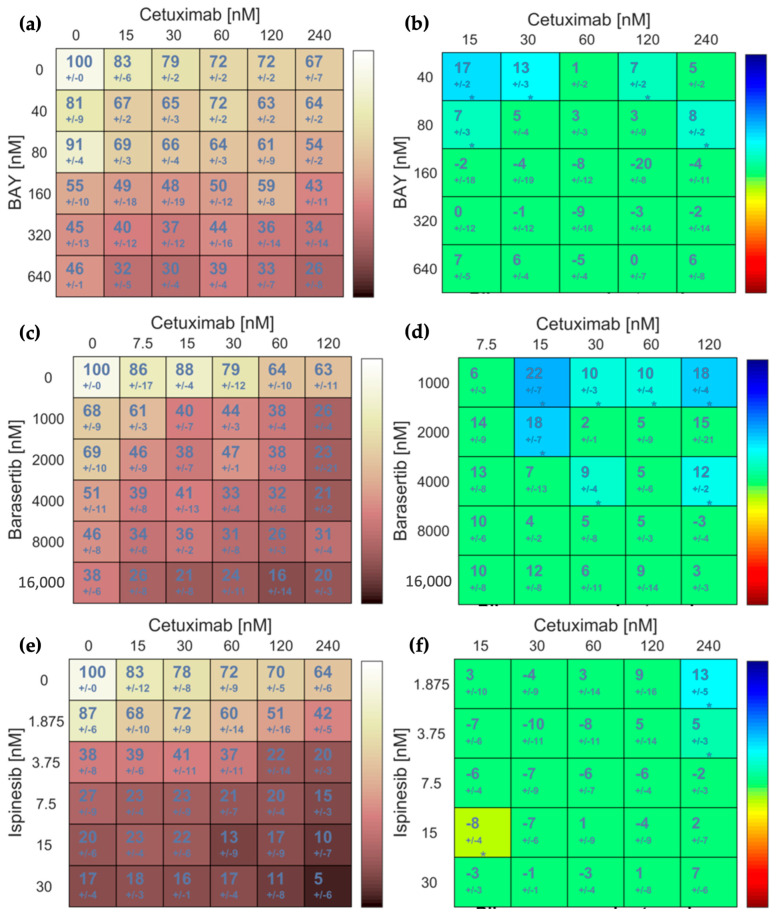
BAY1217389 + Cetuximab (**a**,**b**), Barasertib + Cetuximab (**c**,**d**), and Ispinesib + Cetuximab (**e**,**f**) combinations potentiate cytotoxicity in SCC-25 cell lines. Cell viability (%) after 48 h of exposure to single or combination therapies was determined by MTT assay (**a**,**c**,**e**) based on three independent experiments. Synergy scores (**b**,**d**,**f**) were calculated using the Bliss model in Combenefit software version 2.021, with asterisks denoting synergistic (cyan to blue) or antagonistic (yellow-green to red) effects. The statistical significance levels were the following: * *p* < 0.05.

Given that the SCC-25 cell line serves as a model of oral cancer and exhibits elevated protein expression levels of EGFR, MPS-1, Aurora-B, and KSP compared to SCC-09 cells, we selected SCC-25 cells for the subsequent experiments in this study. The results of the MTT assay for the SCC-09 cell lines can be found in the Supplementary Material (Figure S1). The effects of each drug combination were assessed, and the data are presented as two dual-drug concentration crosswise matrices: one depicting the percentage of cell viability and the other illustrating the effect score of the combinations (Figure 7). Notably, all combinations exhibited synergistic effects, and the synergistic combination with the lowest concentrations was selected for further experimentation (30 nM of Cetuximab with 40 nM of BAY1217389, 15 nM of Cetuximab with 1000 nM of Barasertib, and 240 nM of Cetuximab with 1.875 nM of Ispinesib).

Notably, the concentrations of BAY1217389, Barasertib, and Ispinesib used correspond to 10-, 5.6-, and 3-fold reductions, respectively, from their respective IC_50_ values. In contrast, the reduction in concentration for Cetuximab was even more pronounced, as the IC_50_ could not be achieved.

**Figure 8:**
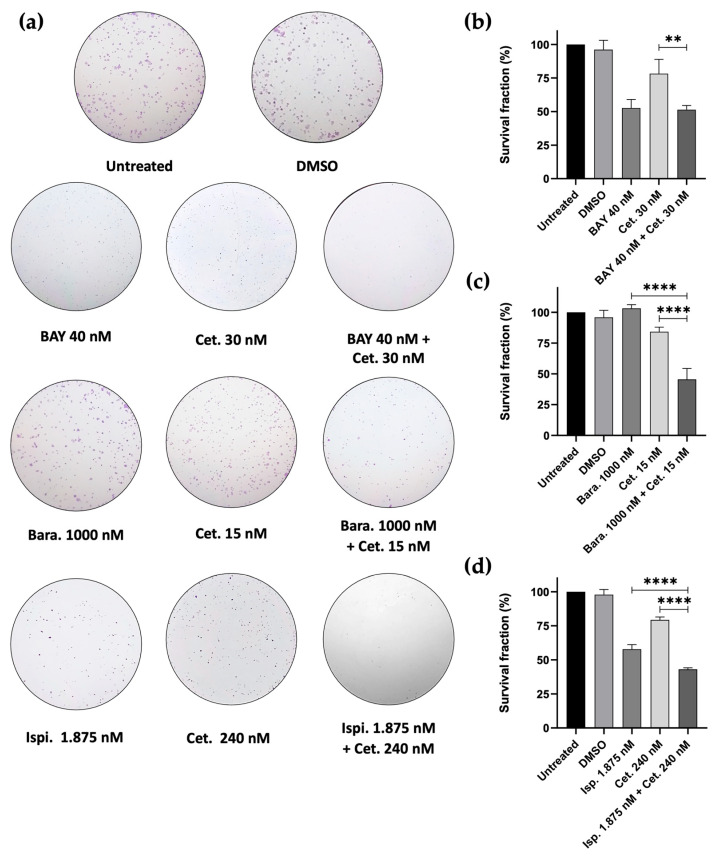
Colony formation assays were conducted using SCC-25 cells over a period of 7 days (**a**). The survival fraction (%) was quantified following single or combination treatments as specified (**b**–**d**). Data represent the mean ± SD of three independent experiments, analyzed using one-way ANOVA followed by Tukey’s multiple comparisons test. Statistical significance is denoted as follows: ** *p* < 0.01; **** *p* < 0.0001.

To evaluate whether drug combinations resulted in prolonged anticancer effects, we performed colony formation assays in SCC-25 cancer cells. The cells were exposed to the different combinations for 48 h, after which the medium was replaced with fresh medium. Colonies were counted after 7 days. Our results indicated that the combinations of Barasertib + Cetuximab and Ispinesib + Cetuximab significantly reduced colony formation compared to single treatments. In contrast, the combination of BAY1217389 + Cetuximab resulted in a reduction similar to that of BAY1217389 alone (Figure 8a–d). Specifically, a colony survival fraction of 53.69% ± 2.64 was observed following treatment with Barasertib + Cetuximab, compared to Barasertib (103.24% ± 3.02) and Cetuximab (84.22% ± 3.68) alone. Similarly, the Ispinesib + Cetuximab combination results in a colony survival fraction of 43% ± 1.18, compared to Ispinesib (57.82% ± 3.32) and Cetuximab (79.37% ± 2.12) drugs alone. In contrast, the combinatorial exposure to BAY1217389 + Cetuximab led to a colony survival fraction of 51.37% ± 3.16 compared to 52.64% ± 6.36 for BAY1217389 monotherapy and 78.25% ± 10.63 for Cetuximab monotherapy. These results suggest that the combinatorial approaches, at least for Barasertib + Cetuximab and Ispinesib + Cetuximab combinations, exhibit the ability to maintain long-term cellular cytotoxicity, preventing the proliferation of cancer cells, supporting the therapeutic promise of combining EGFR inhibition with Aurora-B or KSP inhibition, and to a lesser extent with MPS-1 inhibition, in oral cancer treatment. Thus, these combinations appear to be viable strategies to enhance therapeutic outcomes.

### 3.5. The Combined Treatment of Cetuximab with BAY1217389, Barasertib, or Ispinesib Enhances Mitotic Cell Death in Oral Cancer Cells

After observing increased cytotoxicity with the combination treatments, we further investigated whether this effect was mediated by Cetuximab promoting apoptosis, using flow cytometry for analysis. Cetuximab alone significantly increased the percentage of apoptotic cells compared to the control. BAY1217389 alone demonstrated a slight increase in apoptotic cells compared to the control (7.48 ± 1.60% vs. 2.3 ± 0.72%, respectively), while Barasertib alone resulted in a similar increase as Cetuximab alone (12.2 ± 2.47% vs. 11.63 ± 3.76%, respectively). For the combination of Cetuximab and Ispinesib, cells were exposed for 24 h to ensure the analysis captured all cells undergoing apoptosis, as this combination resulted in a high number of dead cells at this time, thereby minimizing the risk of loss. However, Ispinesib alone did not exhibit a statistically significant difference compared to the control (5.04 ± 1.77% vs. 2.3 ± 1%).

**Figure 9:**
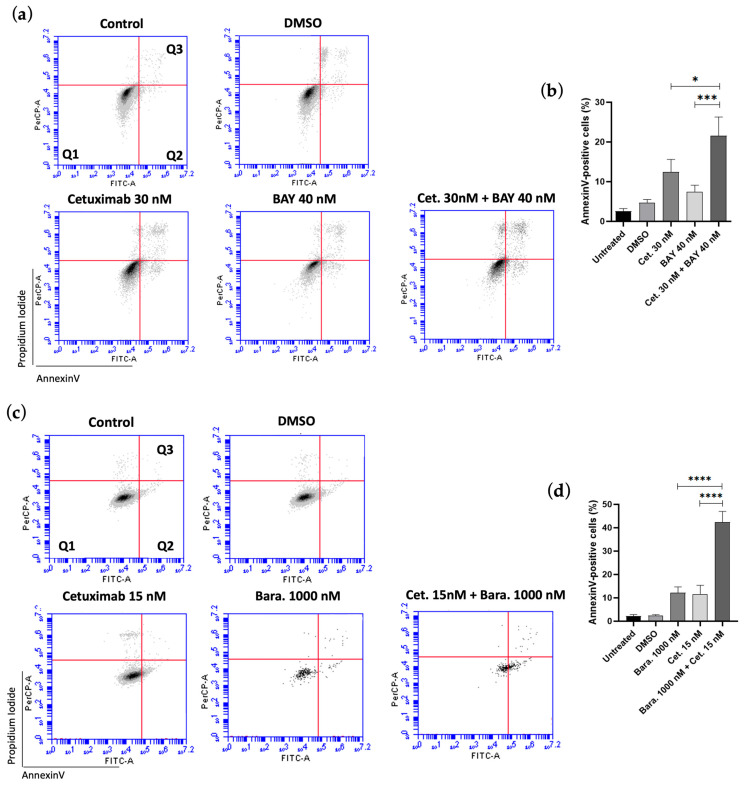

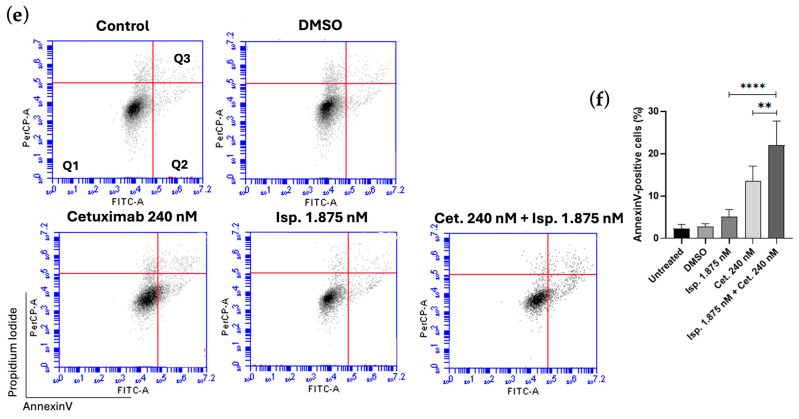
The combinations of BAY1217389 + Cetuximab, Barasertib + Cetuximab, and Ispinesib + Cetuximab promote increased cell death in SCC-25 oral cancer cells. Representative cytograms of SCC-25 cells double-stained with Annexin V-FITC and propidium iodide (PI) are displayed in panels (**a**,**c**,**e**). The quadrants are defined as follows: Q1 = live cells (Annexin V-negative, PI-negative), Q2 = early apoptosis (Annexin V-positive, PI-negative), and Q3 = late apoptosis (Annexin V-positive, PI-positive). Bar graphics (**b**,**d**,**f**) showing the percentage of Annexin V-positive cells. Data represent the mean ± SD from three independent experiments and were analyzed using one-way ANOVA with Tukey’s multiple comparison test. Statistical significance is indicated as * *p* < 0.05, ** *p* < 0.01, *** *p* < 0.001, and **** *p* < 0.0001.

A significant increase in cell apoptosis was observed when Cetuximab was combined with BAY1217389 (21.62 ± 4.70%), Barasertib (42.37 ± 4.61%), or Ispinesib (22 ± 5.75%), compared to the effects of the individual drugs and the control (Figure 9). Additionally, the combinations of Cetuximab with Barasertib or Ispinesib were tested using SCC-09 cells. The combination of Cetuximab and Ispinesib demonstrated similar results, while Cetuximab and Barasertib showed a slight increase, although not statistically significant, in the percentage of apoptotic cells (Figure S2).

## 4. Discussion

EGFR, MPS-1, Aurora-B, and KSP are highly expressed in oral cancer cells and tissues from OSCC patients, which is in accordance with the data available in bioinformatic databases, such as UALCAN. Furthermore, EGFR, MPS-1, Aurora-B, and KSP expression levels have significant implications for the clinical outcomes of OSCC patients. High EGFR expression is associated with poorer prognosis and reduced survival, as indicated by the significant correlation with cancer-specific survival. This makes EGFR a valuable prognostic marker and a target for therapeutic intervention.

While the direct correlation of MPS-1, Aurora-B, and KSP with clinical outcomes, such as survival, was not statistically significant in the provided data, there is a notable trend.

Except for MPS-1, high expression levels of these proteins may correlate with more aggressive disease and poorer prognosis. However, a limitation of our study is the relatively small number of patient samples; a larger sample size might yield statistically significant results, thereby strengthening the validity of our results regarding the overexpression of MPS-1, Aurora-B, and KSP in clinical contexts.

To our knowledge, no studies have specifically assessed the correlation between MPS-1 expression and overall survival in OSCC patients. However, some studies conducted with triple-negative breast cancer (TNBC) patient samples have shown similar results to those observed in our study, wherein high expression of MPS-1 was correlated with better overall survival and disease-free survival [11,12]. One of these studies suggests that low expression of MPS-1 may be associated with reduced responsiveness to conventional chemotherapy, as these cells exhibit lower proliferative rates [11]. Conversely, other studies indicate that higher expression of MPS-1 in TNBC patients is linked to poor prognosis [41,42]. Thus, additional involving OSCC patients is required to fully elucidate the correlation between this protein and patient prognosis.

The trend observed for KSP appears to align with previously reported findings, which indicated that high expression of KSP is associated with worse prognosis in oral cancer patients [39]. Similar results have been reported for Aurora-B, although the correlation with poorer prognosis in OSCC patients was noted in relation to disease-free survival rather than overall survival, as indicated in our study [17].

Co-treatment with Cetuximab and inhibitors targeting MPS-1, Aurora-B, or KSP demonstrated synergistic effects in oral cancer cells. This synergy suggests that these proteins may be part of interconnected pathways that promote cancer cell survival and proliferation. By simultaneously targeting multiple nodes within these pathways, combination treatment may more effectively induce mitotic cell death.

Combining Cetuximab with BAY1217389 enhances mitotic cell death, indicating that inhibiting EGFR and MPS-1 disrupts critical signaling required for cell division and survival in oral cancer cells. Nonetheless, the results regarding the apoptosis evaluation had a more pronounced effect on the combination when compared to the long-term proliferation assay. This can be explained by the inherent differences between the methods used for the experiments, such as the duration of the assay (48 h vs. 7 days). Combining Cetuximab with Barasertib also amplifies mitotic cell death, suggesting that Aurora-B is a crucial mediator of cell cycle progression in these cells, and its inhibition, alongside EGFR blockade, leads to heightened cell death. Similarly to the other combinations, Cetuximab with Ispinesib increases mitotic cell death, indicating the essential role of KSP in mitotic spindle formation and function, which is critical for cell division. In addition to the body of work regarding the clinical relevance of these proteins, the results from our combinatorial treatment approaches suggest their potential as therapeutic targets.

Furthermore, the cell lines employed in this study were both HPV-negative SCCs from the tongue. HPV-negative oral cancers are generally associated with a poorer prognosis compared to their HPV-positive counterparts. Additionally, these two types exhibit distinct characteristics that must be considered when selecting treatment options. Therefore, our findings may not be extrapolated to HPV-positive cell lines, even though previous research has demonstrated similar responses to Cetuximab treatment regardless of HPV status [43]. Moreover, it is important to note that the incidence of HPV-positive oral cancers is on the rise, particularly in developed countries. This trend highlights the necessity for further research that includes HPV-positive cell lines to enhance our understanding of the effects of our combinatorial treatment approaches.

Our findings indicate that MPS-1, Aurora-B, or KSP are commonly expressed in OSCC, and inhibiting these proteins enhances the therapeutic potential of Cetuximab.

## 5. Conclusions

The overexpression of EGFR, MPS-1, Aurora-B, and KSP in OSCC underscores their critical roles in tumor progression. The synergistic effects of combined Cetuximab, and specific inhibitors highlight the potential for multi-targeted therapies. EGFR, with its significant association with poor prognosis, stands out as a key prognostic marker and therapeutic target. Future studies should aim to validate these findings in larger patient cohorts and further explore the interplay mechanism underlying the observed synergistic effects. This could pave the way for more effective, personalized treatment strategies for OSCC patients.

## Figures and Tables

**Table 1:** Clinicopathological characteristics of the OSCC patients and their association with EGFR and MPS-1 staining intensity.

		EGFR (N = 29)			MPS-1 (N = 25)		
		Staining Intensity			Staining Intensity		
		Low	High		Low	High	
**Characteristic**	***N* (%)**	***N* (%)**	***N* (%)**	***p ^a^***	***N* (%)**	***N (%)***	***p ^a^***
All cases	30						
**Gender**							
Female	7 (23.3)	3 (16.7)	4 (36.4)	0.229	2 (40)	4 (20)	0.349
Male	23 (76.7)	15 (83.3)	7 (63.6)		3 (60)	16 (80)	
**Age**							
<62 years	12 (40)	5 (27.8)	7 (63.6)	0.057	2 (40)	8 (40)	1.000
≥62 years	18 (60)	13 (72.2)	4 (36.4)		3 (60)	12 (60)	
**Tumor location**							
Lip	6 (20)	6 (33.3)	0 (0)	0.177	1 (20)	2 (10)	0.619
Floor of the mouth	4 (13.3)	2 (11.1)	2 (11.1)		1 (20)	3 (15)	
Tongue	10 (33.3)	5 (27.8)	4 (36.4)		3 (60)	5 (25)	
Buccal mucosa	2 (6.7)	1 (5.6)	1 (9.1)		0 (0)	2 (10)	
Retromolar trigone	2 (6.7)	2 (11.1)	0 (0)		0 (0)	2 (10)	
Hard palate	4 (13.3)	2 (11.1)	2 (11.1)		0 (0)	4 (20)	
Alveolar ridge	2 (6.7)	0 (0)	2 (11.1)		0 (0)	2 (10)	
**Stage**							
I + II	18 (60)	12 (66.7)	5 (45.5)	0.260	3 (60)	11 (55)	0.840
III + IV	12 (40)	6 (33.3)	6 (54.4)		2 (40)	9 (45)	
**Treatment modality**							
SG	17 (56.7)	11 (61.1)	5 (45.5)	0.411	3 (60)	11 (55)	0.840
SG + RT	13 (43.3)	7 (38.9)	6 (54.5)		2 (40)	9 (45)	
**Tumor Grade**							
G1	18 (60)	11 (61.1)	6 (54.5)	0.728	3 (60)	11 (55)	0.840
G2 + G3	12 (40)	7 (38.9)	5 (45.5)		2 (40)	9 (45)	
**Margin status**							
Free of tumor	19 (63.3)	12 (75)	6 (60)	0.420	3 (60)	13 (68.4)	0.722
Tumor proximity and with tumor	8 (26.7)	4 (25)	4 (40)		2 (40)	6 (31.6)	
**Vascular invasion**							
Absent	29 (96.7)	17 (94.4)	11 (100)	0.426	5 (100)	19 (95)	0.610
Present	1 (3.3)	1 (5.6)	0 (0)		0 (0)	1 (5)	
**Perineural permeation**							
Absent	26 (86.7)	16 (88.9)	9 (81.8)	0.592	4 (80)	17 (85)	0.785
Present	4 (13.3)	2 (11.1)	2 (18.2)		1 (20)	3 (15)	
**Lymphatic invasion**							
Absent	24 (80)	16 (88.9)	7 (63.6)	0.103	4 (80)	15 (75)	0.815
Present	6 (20)	2 (11.1)	4 (36.4)		1 (20)	5 (25)	
**Muscular invasion**							
Absent	25 (83.8)	14 (77.8)	10 (90.9)	0.364	4 (80)	16 (80)	1.000
Present	5 (16.7)	4 (22.2)	1 (9.1)		1 (20)	4 (20)	

*^a^* Chi-square test. *p* values with statistically significant differences highlighted in bold (*p* < 0.05).

**Table 2:** Clinicopathological characteristics of the OSCC patients and their association with Aurora-B extent and staining intensity.

	AurB (N = 20)					
	Extent			Staining Intensity		
	≤9%	≥10%		Low	High	
**Characteristic**	***N* (%)**	***N* (%)**	***p ^a^***	***N* (%)**	***N* (%)**	***p ^a^***
All cases						
**Gender**						
Female	4 (36.4)	2 (22.2)	0.492	4 (36.4)	2 (22.2)	0.492
Male	7 (63.6)	7 (77.8)		7 (63.6)	7 (77.8)	
**Age**						
<62 years	4 (36.4)	5 (55.6)	0.391	4 (36.4)	5 (55.6)	0.391
≥62 years	7 (63.6)	4 (44.4)		7 (63.6)	4 (44.4)	
**Tumor location**						
Lip	1 (9.1)	1 (11.1)	0.311	1 (9.1)	1 (11.1)	0.311
Floor of the mouth	1 (9.1)	2 (22.2)		1 (9.1)	2 (22.2)	
Tongue	6 (54.5)	1 (11.1)		6 (54.5)	1 (11.1)	
Buccal mucosa	1 (9.1)	1 (11.1)		1 (9.1)	1 (11.1)	
Retromolar trigone	0 (0)	1 (11.1)		0 (0)	1 (11.1)	
Hard palate	2 (18.2)	1 (11.1)		2 (18.2)	1 (11.1)	
Alveolar ridge	0 (0)	2 (22.2)		0 (0)	2 (22.2)	
**Stage**						
I + II	7 (63.6)	4 (44.4)	0.391	7 (63.6)	4 (44.4)	0.391
III + IV	4 (36.4)	5 (55.6)		4 (36.4)	5 (55.6)	
**Treatment modality**						
SG	6 (54.5)	5 (55.6)	0.964	6 (54.5)	5 (55.6)	0.964
SG + RT	5 (45.5)	4 (44.4)		5 (45.5)	4 (44.4)	
**Tumor Grade**						
G1	7 (63.6)	6 (66.7)	0.888	7 (63.6)	6 (66.7)	0.888
G2 + G3	4 (36.4)	3 (33.3)		4 (36.4)	3 (33.3)	
**Margin status**						
Free of tumor	6 (54.5)	7 (77.8)	0.279	6 (54.5)	7 (77.8)	0.279
Tumor proximity and with tumor	5 (45.5)	2 (22.2)		5 (45.5)	2 (22.2)	
**Vascular invasion**						
Absent	11 (100)	9 (100)	-	11 (100)	9 (100)	-
Present	0 (0)	0 (0)		0 (0)	0 (0)	
**Perineural permeation**						
Absent	9 (81.8)	8 (88.9)	0.660	9 (81.8)	8 (88.9)	0.660
Present	2 (18.2)	1 (11.1)		2 (18.2)	1 (11.1)	
**Lymphatic invasion**						
Absent	9 (81.8)	6 (66.7)	0.436	9 (81.8)	6 (66.7)	0.436
Present	2 (18.2)	3 (33.3)		2 (18.2)	3 (33.3)	
**Muscular invasion**						
Absent	9 (81.8)	8 (88.9)	0.660	9 (81.8)	8 (88.9)	0.660
Present	2 (18.2)	1 (11.1)		2 (18.2)	1 (11.1)	

*^a^* Chi-square test. *p* values with statistically significant differences highlighted in bold (*p* < 0.05).

**Table 3:** Clinicopathological characteristics of the OSCC patients and their association with KSP extent and staining intensity.

	KSP (N = 20)					
	Extent			Staining Intensity		
	≤9%	≥10%		Low	High	
**Characteristic**	***N* (%)**	***N* (%)**	***p ^a^***	***N* (%)**	***N* (%)**	***p ^a^***
All cases						
**Gender**						
Female	1 (25)	5 (31.2)	0.807	4 (25)	2 (50)	0.329
Male	3 (75)	11 (68.8)		12 (75)	2 (50)	
**Age**						
<62 years	3 (75)	6 (37.5)	0.178	7 (43.8)	2 (50)	0.822
≥62 years	1 (25)	10 (62.5)		9 (56.2)	2 (50)	
**Tumor location**						
Lip	1 (25)	4 (25)	0.868	3 (18.8)	2 (50)	0.517
Floor of the mouth	1 (25)	1 (6.3)		1 (6.2)	1 (25)	
Tongue	1 (25)	5 (31.1)		6 (37.5)	0 (0)	
Buccal mucosa	0 (0)	2 (12.5)		2 (12.5)	0 (0)	
Retromolar trigone	0 (0)	1 (6.3)		1 (6.2)	0 (0)	
Hard palate	1 (25)	2 (12.5)		2 (12.5)	1 (25)	
Alveolar ridge	0 (0)	1 (6.3)		1 (6.2)	0 (0)	
**Stage**						
I + II	3 (75)	7 (43.8)	0.264	7 (43.8)	3 (75)	0.264
III + IV	1 (25)	9 (56.2)		9 (56.2)	1 (25)	
**Treatment modality**						
SG	2 (50)	8 (50)	1.000	8 (50)	2 (50)	1.000
SG + RT	2 (50)	8 (50)		8 (50)	2 (50)	
**Tumor Grade**						
G1	1 (25)	9 (56.2)	0.264	7 (43.8)	3 (75)	0.264
G2 + G3	3 (75)	7 (43.8)		9 (56.2)	1 (25)	
**Margin status**						
Free of tumor	2 (50)	9 (69.2)	0.482	9 (64.3)	2 (66.7)	0.938
Tumor proximity and with tumor	2 (50)	4 (30.8)		5 (35.7)	1 (33.3)	
**Vascular invasion**						
Absent	4 (100)	16 (100)	-	16 (100)	4 (100)	-
Present	0 (0)	0 (0)		0 (0)	0 (0)	
**Perineural permeation**						
Absent	3 (75)	15 (93.8)	0.264	14 (87.5)	4 (100)	0.456
Present	1 (25)	1 (6.2)		2 (12.5)	0 (0)	
**Lymphatic invasion**						
Absent	3 (75)	13 (81.2)	0.780	12 (75)	4 (100)	0.264
Present	1 (25)	3 (18.8)		4 (25)	0 (0)	
**Muscular invasion**						
Absent	4 (100)	16 (100)	-	16 (100)	4 (100)	-
Present	0 (0)	0 (0)		0 (0)	0 (0)	

*^a^* Chi-square test. *p* values with statistically significant differences highlighted in bold (*p* < 0.05).

**Table 4:** Univariate analysis of cancer-specific survival (CSS) according to the clinicopathological characteristics and expression of EGFR, MPS-1, Aurora-B, and KSP.

Characteristic	*N* (%)	Dead	CSS *^a^*	*p ^b^*
All cases	30			
**Stage**				
I	7 (23.3)	0	0	**0.020**
II	11 (36.7)	4	72.7	
III	5 (16.7)	1	80	
IV	7 (23.3)	5	28.6	
**Treatment modality**				
SG	17 (56.7)	3	87.5	**0.027**
SG + RT	13 (43.3)	7	44.9	
**Tumor grade**				
G1	18 (60)	5	75.5	0.346
G2 + G3	12 (40)	5	58.3	
**Vascular invasion**				
Absent	29 (96.7)	10	67.3	0.534
Present	1 (3.3)	0	0	
**Perineural permeation**				
Absent	26 (86.7)	8	71.4	0.231
Present	4 (13.3)	2	50	
**Lymphatic invasion**				
Absent	24 (80)	8	69.1	0.729
Present	6 (20)	2	66.7	
**Muscular invasion**				
Absent	25 (83.3)	9	65.9	0.482
Present	5 (16.7)	1	80	
**EGFR staining intensity**				
Low	19 (63.3)	4	76.4	**0.023**
High	11 (36.7)	7	43.6	
**MPS-1 staining intensity**				
Low	5 (20)	3	30	0.102
High	20 (80)	5	80	
**AurB staining intensity**				
Low	11 (55)	4	63.6	0.683
High	9 (45)	4	62.2	
**KSP staining intensity**				
Low	16 (80)	5	67	0.335
High	4 (20)	3	50	

*^a^* Percentage of cases without event at 3 years of follow-up. *^b^* Log-rank test. Information not available for every patient. *p* values with statistically significant differences are highlighted in bold (*p* < 0.05).

**Table 5:** Multivariate analysis of cancer-specific survival (CSS).

Variables		Exp (B)	*p*
**Type of treatment**	Surgery	1	
	Surgery + other treatments	2.066 (0.236–18.040)	0.512
**EGFR intensity score**	0/low/moderate	1	
	strong	4.745 (1.170–19.241)	**0.029**
**Stage**	I + II	1	
	III + IV	1.599 (0.602–4.244)	0.346

*p* values with statistically significant differences highare lighted in bold (*p* < 0.05).

**Table 6:** IC_50_ values of Cetuximab, BAY1217389, Barasertib, and Ispinesib in SCC-25 and SCC-09 cell lines after 48 h incubation.

	IC_50_ (nM)	
Drugs/Cell Line	SCC-25	SCC-09
Cetuximab	>800	>800
BAY1217389	402.95 ± 4.31	540.6 ± 2.12
Barasertib	5580.0 ± 664.0 [40]	>64,000.0 [40]
Ispinesib	3.4 ± 0.5 [40]	58.9 ± 3.2 [40]

## Data Availability

Data can be shared upon request.

## Supplementary Materials

The following supporting information can be downloaded at: https://www.mdpi.com/article/10.3390/cancers16223732/s1, Table S1: Clinicopathological characteristics of the OSCC patients; Figure S1: BAY1217389 + Cetuximab (a,b), Barasertib + Cetuximab (c,d) and Ispinesib + Cetuximab (e,f) combinations potentiate cytotoxicity in SCC-09 cell lines. Cell viability (%) of single or combination therapies after 48 h of drug exposure, from three independent experiments as determined by MTT assay (a,c,d). Synergy scores (b,d,f) calculated by the Bliss model of Combenefit software 2.021 with statistical relevance of * *p* < 0.05; Figure S2: The combination of Barasertib + Cetuximab (a,b) showed no cell death increase while Ispinesib + Cetuximab (c,d) enhanced cell death in SCC-09 oral cancer cells. Representative cytograms of the SCC-09 cell line, double-stained with Annexin V-FITC and propidium iodide (PI), are shown (a,c). The quadrants are defined as follows: Q1 = live cells (Annexin V-negative and PI-negative), Q2 = early stage of apoptosis (Annexin V-positive and PI-negative), and Q3 = late stage of apoptosis (Annexin V-positive and PI-positive). Quantification of Annexin V-positive cells is provided (b,d). Data represent the mean ± SD of three independent experiments and were analyzed using one-way ANOVA followed by Tukey’s multiple comparisons test. Statistical significance is indicated as ** *p* < 0.01, and *** *p* < 0.001. File S1: Original western blots.
