# Supplementary material for: Targeting the EGFR and Spindle Assembly Checkpoint Pathways in Oral Cancer: A Plausible Alliance to Enhance Cell Death
Source: Cancers (Basel). 2024 Nov 5;16(22):3732. doi: 10.3390/cancers16223732 (PMC11591835; doi:10.3390/cancers16223732)
Supplement: Supplementary file 1 [file cancers-16-03732-s001.zip › cancers-3233056-supplementary.pdf]

Supplementary File

Table S1. Clinicopathological characteristics of the OSCC patients.

Characteristic	*N* (%)
All cases	30
**Gender**	
Female	7 (23.3)
Male	23 (76.7)
**Age**	
<62 years	12 (40)
≥62 years	18 (60)
**Tumor location**	
Lip	6 (20)
Floor of the mouth	4 (13.3)
Tongue	10 (33.3)
Buccal mucosa	2 (6.7)
Retromolar trigone	2 (6.7)
Hard palate	4 (13.3)
Alveolar ridge	2 (6.7)
**Stage**	
I + II	18 (60)
III + IV	12 (40)
**Treatment modability**	
SG	17 (56.7)
SG + RT	13 (43.3)
**Tumor Grade**	
G1	18 (60)
G2 + G3	12 (40)
**Margin status**[a]	
Free of tumor	19 (63.3)
Tumor proximity and with tumor	8 (26.7)
**Vascular invasion**	
Absent	29 (96.7)
Present	1 (3.3)
**Perineural permeation**	
Absent	26 (86.7)
Present	4 (13.3)
**Lymphatic invasion**	
Absent	24 (80)
Present	6 (20)

**Muscular invasion**	
Absent	25 (83.3)
Present	5 (16.7)

Abbreviations: SG: surgery; RT: radiotherapy; CT: chemotherapy. [a]Not available in 3 cases.

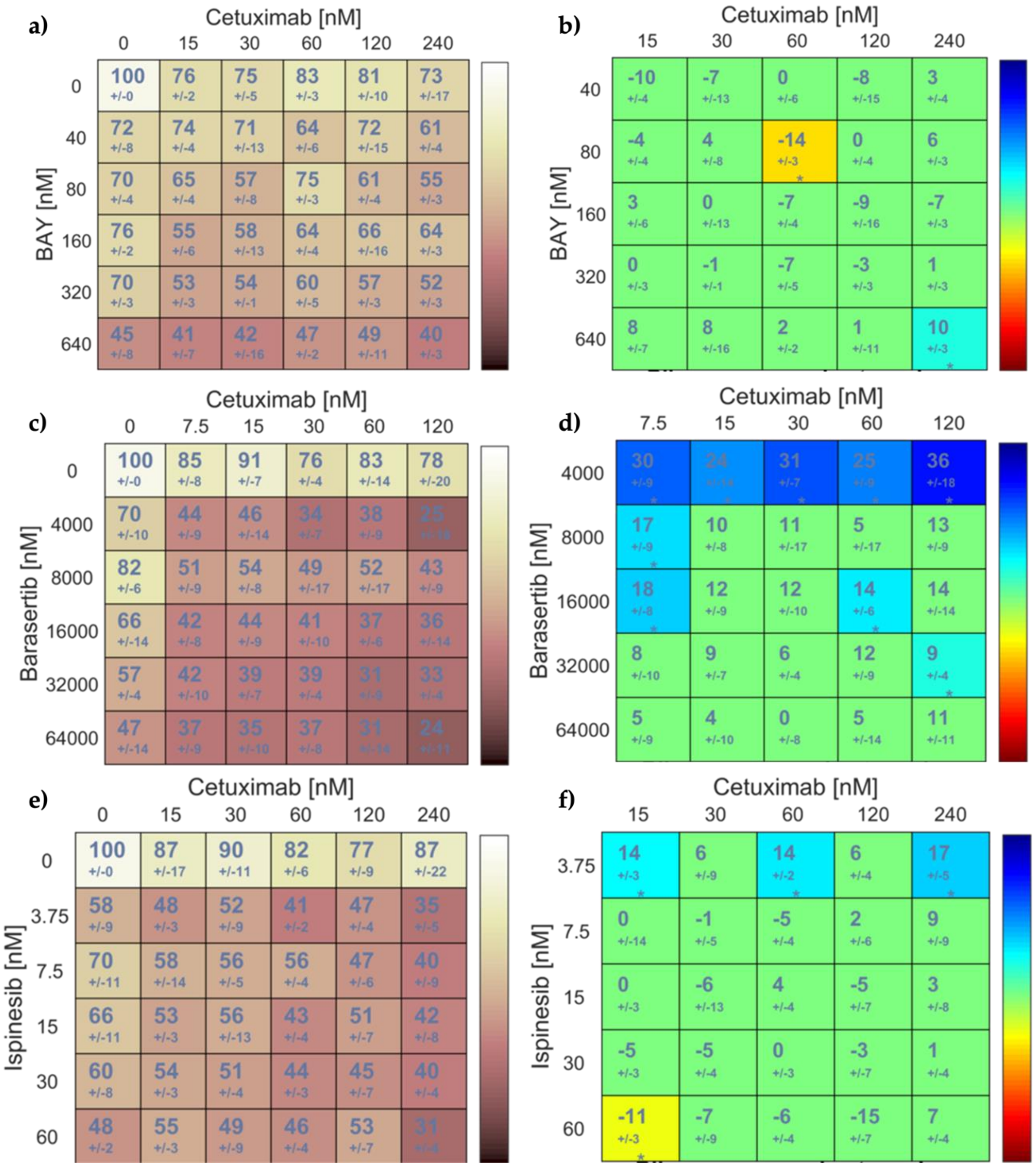

Figure S1. BAY1217389 + Cetuximab (**a, b**), Barasertib + Cetuximab (**c, d**) and Ispinesib + Cetuximab (**e, f**) combinations potentiate cytotoxicity in SCC-09 cell lines. Cell viability (%) of single or combination therapies after 48h of drug exposure, from three independent experiments as determined by MTT assay (**a, c, d**). Synergy scores (**b, d, f**) calculated by the Bliss model of Combenefit software 2.021 with statistical relevance of * $p < 0.05$.

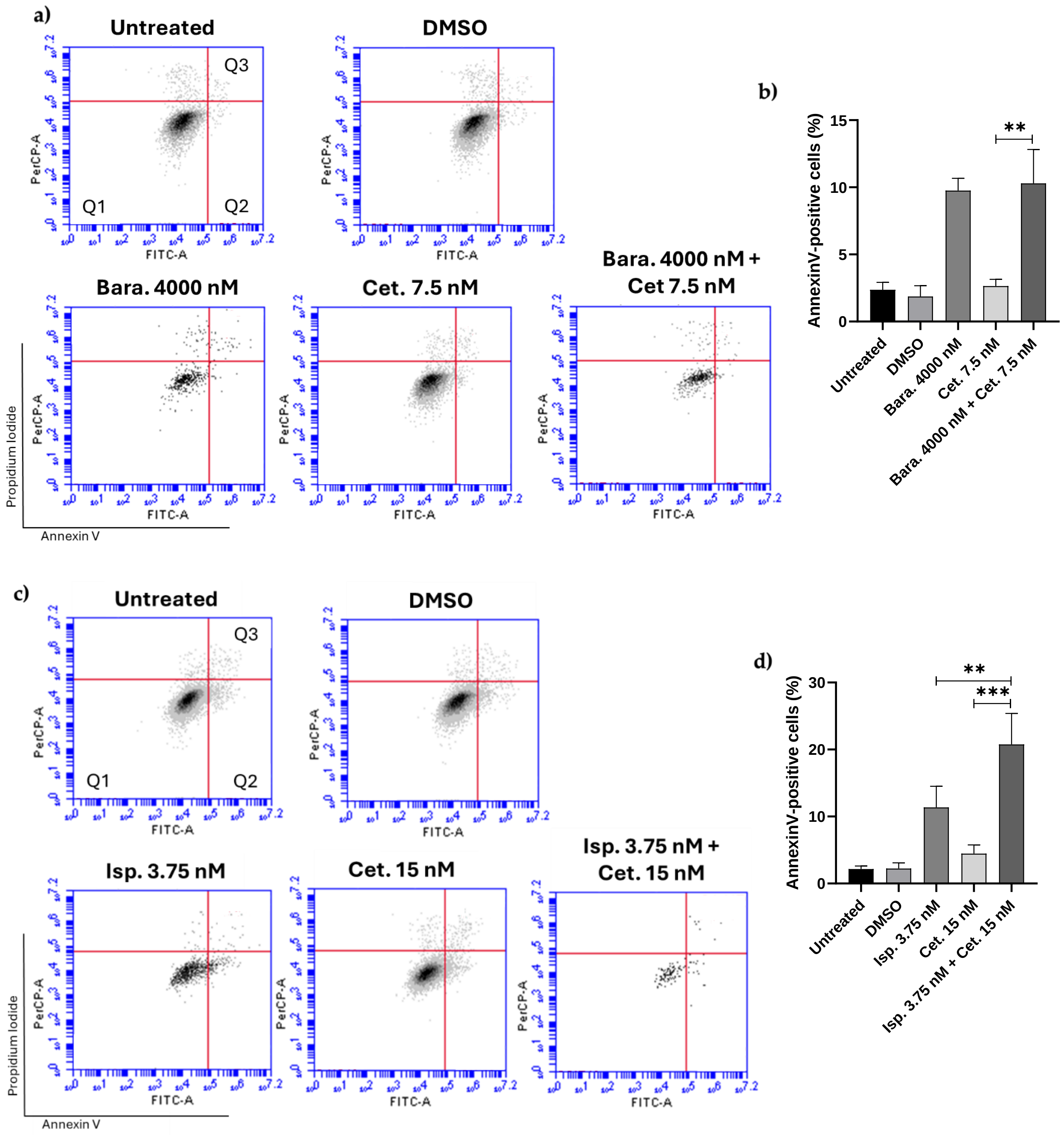

Figure S2. The combination of Barasertib + Cetuximab (**a, b**) showed no cell death increase while Ispinesib + Cetuximab (**c, d**) enhanced cell death in SCC-09 oral cancer cells. Representative cytograms of the SCC-09 cell line, double-stained with Annexin V-FITC and propidium iodide (PI), are shown (a, c). The quadrants are defined as follows: Q1 = live cells (Annexin V-negative and PI-negative), Q2 = early stage of apoptosis (Annexin V-positive and PI-negative), and Q3 = late stage of apoptosis (Annexin V-positive and PI-positive). Quantification of Annexin V-positive cells is provided (b, d). Data represent the mean ± SD of three independent experiments and were analyzed using one-way ANOVA followed by Tukey's multiple comparisons test. Statistical significance is indicated as ** $p < 0.01$, and *** $p < 0.001$.
