# Supplementary figures and images for: Targeting the EGFR and Spindle Assembly Checkpoint Pathways in Oral Cancer: A Plausible Alliance to Enhance Cell Death
Source: Cancers (Basel). 2024 Nov 5;16(22):3732. doi: 10.3390/cancers16223732 (PMC11591835; doi:10.3390/cancers16223732)

EGFR

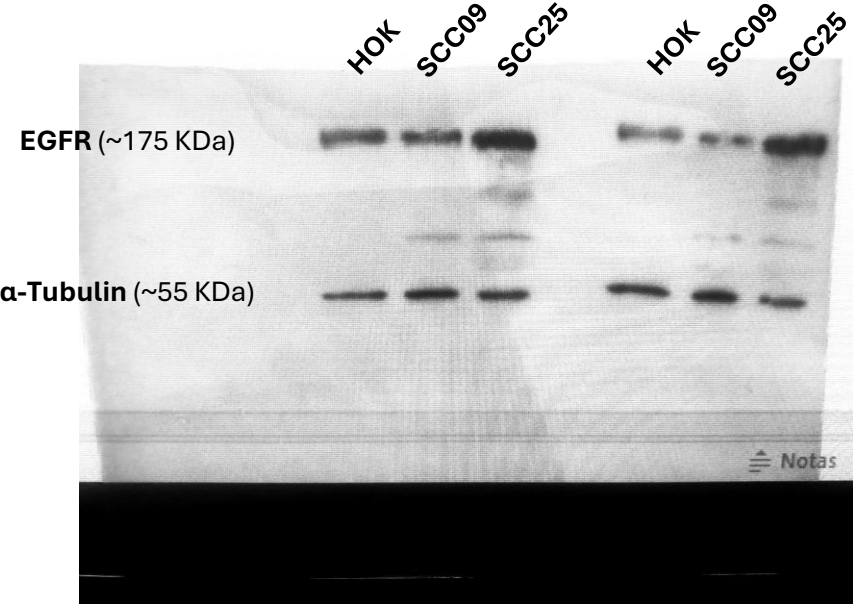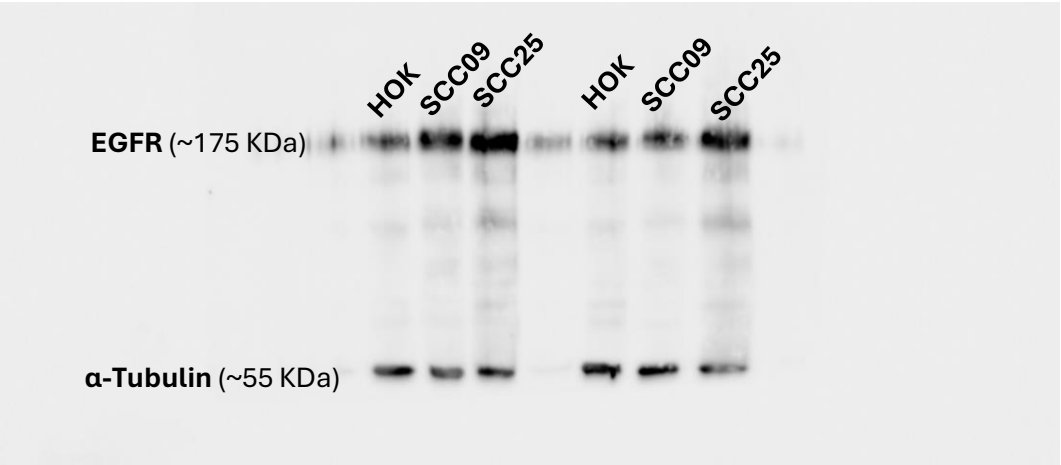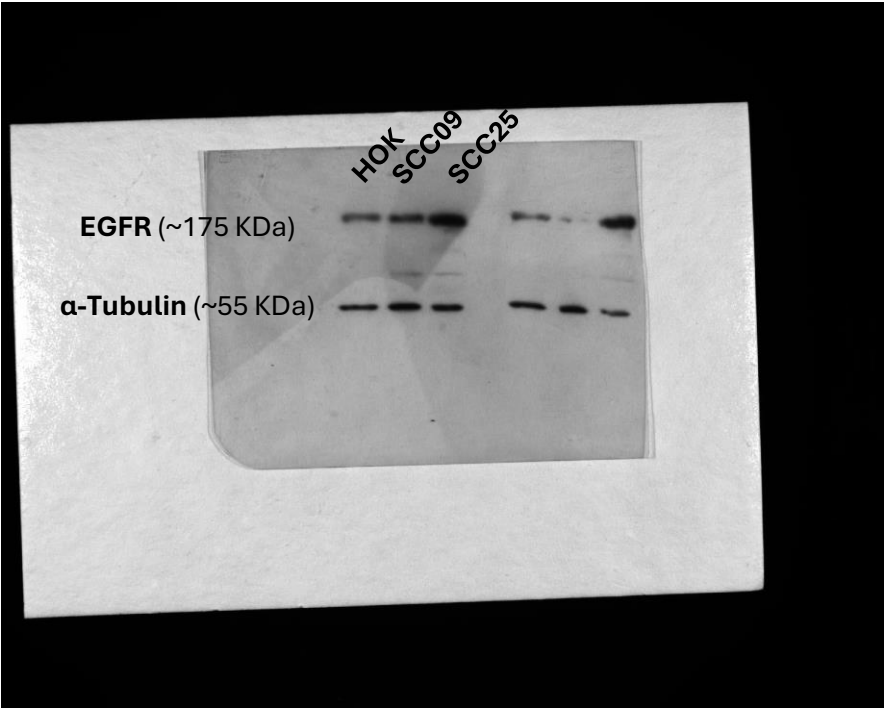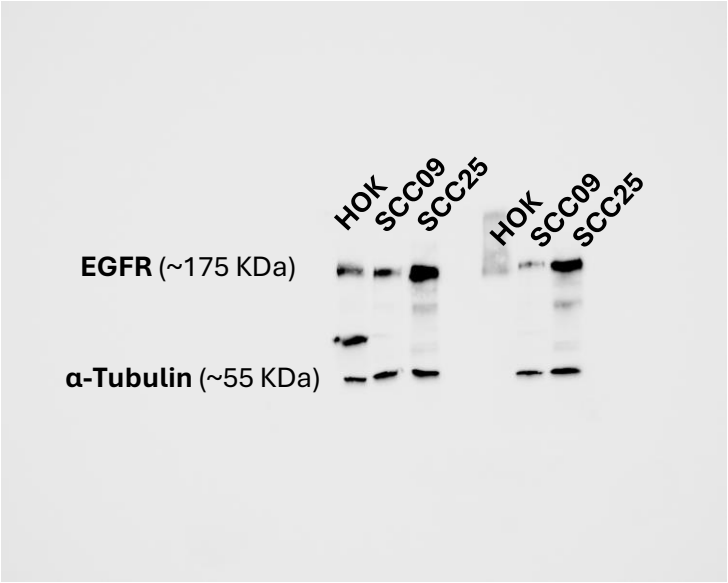

**MPS-1**

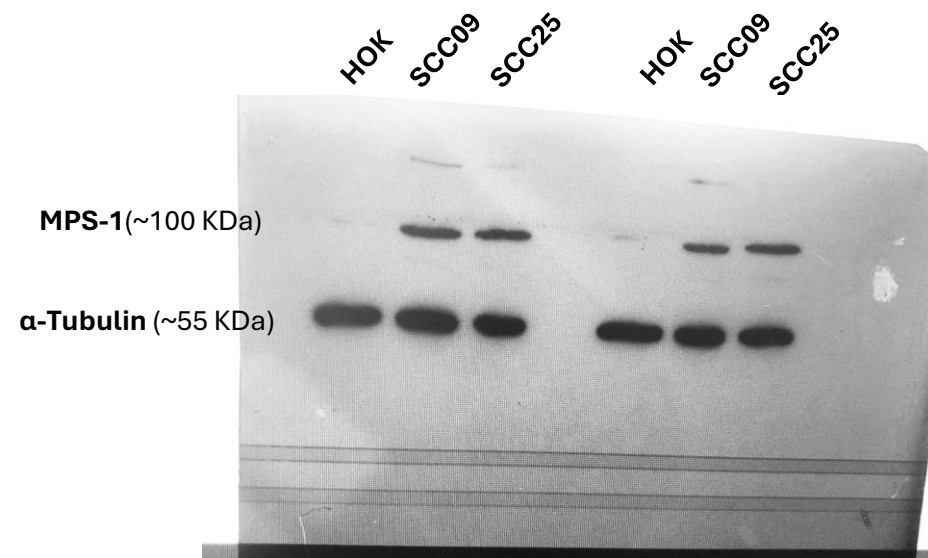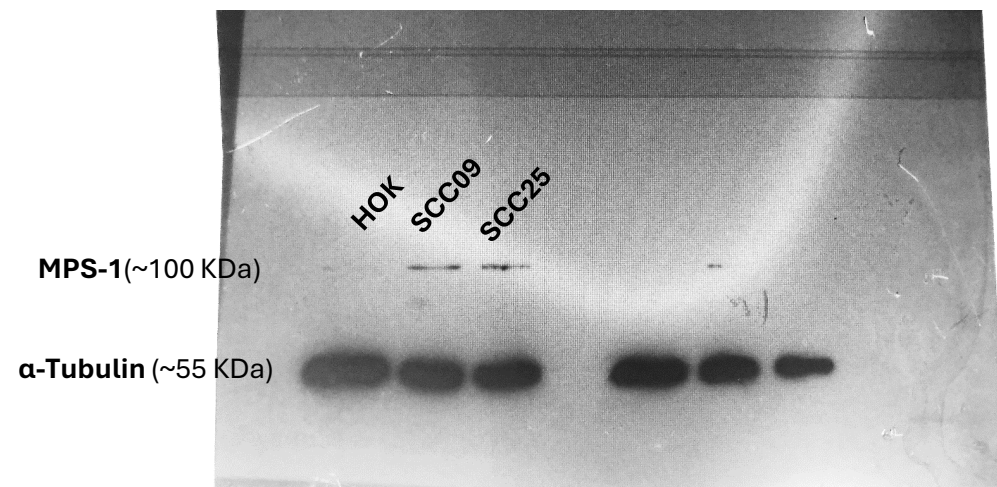

Supplement: Supplementary file 1 [file cancers-16-03732-s001.zip › File S1.pdf]
